# Supplementary material for: Long-term music instruction is partially associated with the development of socioemotional skills
Source: PLoS One. 2024 Jul 18;19(7):e0307373. doi: 10.1371/journal.pone.0307373 (PMC11257369; doi:10.1371/journal.pone.0307373)
Supplement: S1 Table — Sample sizes for each outcome measure. “x” indicates that no data is available for that year for that variable. Phrases two and four for the pitch-matching task and 180-bpm stimuli for the rhythmic entrainment task were not collected at baseline and year 1, respectively. As such, a PCA component was not generated for those years due to the incompleteness of the data. (DOCX) [file pone.0307373.s001.docx]

| Year | Baseline | Year 1 | Year 2 | Year 3 | Year 4 |
| --- | --- | --- | --- | --- | --- |
| Pitch-Matching | x | 62 | 62 | 59 | 50 |
| Rhythmic Entrainment | 56 | x | 64 | 63 | 54 |
| Stickers Away | x | x | 66 | 63 | 56 |
| IECA | 83 | 73 | 67 | 63 | 59 |
| Eyes Test | 83 | 72 | 67 | 64 | 57 |
| Emotion-Matching | 83 | 73 | 67 | 63 | 58 |
