# Supplementary material for: Long-term music instruction is partially associated with the development of socioemotional skills
Source: PLoS One. 2024 Jul 18;19(7):e0307373. doi: 10.1371/journal.pone.0307373 (PMC11257369; doi:10.1371/journal.pone.0307373)
Supplement: S2 Table — Regression model estimates predicting pitch-matching and rhythmic entrainment ability as a function of group, year, and their interaction. The pitch-matching model indicates that an increase in general cognitive ability improves pitch-matching ability by 0.20 points. Likewise, the change in pitch-matching for individuals in the control group decreased significantly over time relative to those in the music group by about 0.26 points (p = 0.016). The rhythmic entrainment model reveals that individuals in the music group scored about 0.70 higher than those in the control group (p = 0.013). Similarly, the music group trended towards outperforming the sports group by about 0.51 points (p = 0.08). Time was the strongest predictor improving rhythmic entrainment by nearly 0.29 points each year (p < .001). (DOCX) [file pone.0307373.s002.docx]

| Effect | *β* | *SE* | *t* | *p* | *95% CI* |
| --- | --- | --- | --- | --- | --- |
| pitch-matching ~ group*year + fsiq4 + (year\|record_id) | | | | | |
| Intercept | -1.552 | 0.660 | -2.350 | 0.020 | [-0.30, 0.51] |
| Control | 0.120 | 0.282 | 0.426 | 0.67 | [-0.47, 0.66] |
| Sport | -0.128 | 0.286 | -0.449 | 0.66 | [-0.67, 0.48] |
| Year | 0.071 | 0.073 | 0.973 | 0.34 | [-0.09, 0.21] |
| FSIQ-4 | 0.017 | 0.006 | 2.603 | 0.010 | [0.06, 0.35] |
| Control x Year | -0.256 | 0.102 | -2.509 | 0.016 | [-0.45, -0.04] |
| Sport x Year | -0.144 | 0.100 | -1.436 | 0.16 | [-0.36, -0.05] |
| rhythmic entrainment ~ group*year + (year\|record_id) | | | | | |
| Intercept | -0.271 | 0.196 | -1.39 | 0.171 | [-0.66, 0.12] |
| Control | -0.700 | 0.275 | -2.439 | 0.013 | [-1.25, -0.15] |
| Sport | -0.512 | 0.286 | -1.792 | 0.077 | [-1.08, 0.06] |
| Year | 0.289 | 0.042 | 6.841 | < 0.001 | [0.21, 0.37] |
| Control x Year | 0.011 | 0.061 | 0.179 | 0.858 | [-0.11, 0.13] |
| Sport x Year | 0.021 | 0.062 | 0.343 | 0.732 | [-0.10, 0.14] |
