# Supplementary material for: Long-term music instruction is partially associated with the development of socioemotional skills
Source: PLoS One. 2024 Jul 18;19(7):e0307373. doi: 10.1371/journal.pone.0307373 (PMC11257369; doi:10.1371/journal.pone.0307373)
Supplement: S3 Table — Regression model estimates predicting socioemotional skills as a function of group, year, and their interaction. While sharing did not vary as a function of these predictors, measures of empathy did, albeit without consistency. The Index of Empathy for Children and Adolescents (IECA) improved by about 0.02 points (p = 0.004) with each unit increase of working memory. Performance on the Eyes Test improved each year by about 0.005 points (p < .001). Finally, performance on the Fiction Emotion-Matching task improved by about 0.05 points each year (p = 0.026), with those in the sports condition performing significantly worse over time at a rate about -0.05 less (p = 0.021) than those in the music group. (DOCX) [file pone.0307373.s003.docx]

| Effect | *β* | *SE* | *t* | *p* | *95% CI* |
| --- | --- | --- | --- | --- | --- |
| Sharing | | | | | |
| Intercept | 0.216 | 0.096 | 2.249 | 0.026 | [0.03, 0.41] |
| Control | -0.021 | 0.132 | -0.162 | 0.871 | [-0.28, 0.24] |
| Sport | -0.191 | 0.134 | -1.420 | 0.158 | [-0.46, 0.07] |
| Year | 0.072 | 0.031 | -2.288 | 0.024 | [0.01, 0.13] |
| Control x Year | -0.040 | 0.043 | 0.916 | 0.361 | [-0.05, 0.13] |
| Sport x Year | 0.090 | 0.044 | 2.054 | 0.042 | [0.00, 0.18] |
| Index of Empathy for Children and Adolescents | | | | | |
| Intercept | 0.529 | 0.025 | 21.600 | < 0.001 | [0.48, 0.58] |
| Control | -0.006 | 0.034 | -0.172 | 0.864 | [-0.07, 0.06] |
| Sport | 0.023 | 0.034 | 0.674 | 0.502 | [-0.04, 0.09] |
| Year | 0.009 | 0.010 | 0.857 | 0.394 | [-0.01, 0.03] |
| Working Memory | 0.018 | 0.006 | 2.907 | 0.004 | [0.01, 0.03] |
| Control x Year | 0.006 | 0.014 | 0.396 | 0.693 | [-0.02, 0.03] |
| Sport x Year | 0.009 | 0.014 | 0.627 | 0.532 | [-0.02, 0.04] |
| Reading the Mind in the Eyes | | | | | |
| Intercept | 0.515 | 0.026 | 19.592 | < 0.001 | [0.46, 0.57] |
| Control | -0.011 | 0.036 | -0.298 | 0.767 | [-0.08, 0.06] |
| Sport | 0.045 | 0.037 | 1.238 | 0.219 | [-0.03, 0.12] |
| Year | 0.045 | 0.008 | 5.692 | < 0.001 | [0.03, 0.06] |
| Control x Year | 0.005 | 0.011 | 0.502 | 0.617 | [-0.02, 0.03] |
| Sport x Year | 0.010 | 0.011 | 0.953 | 0.343 | [-0.01, 0.03] |
| Fiction Emotion-Matching | | | | | |
| Intercept | 0.716 | 0.051 | 13.907 | < 0.001 | [0.61, 0.82] |
| Control | -0.041 | 0.070 | -0.588 | 0.558 | [-0.18, 0.10] |
| Sport | 0.061 | 0.071 | 0.858 | 0.393 | [-0.08, 0.20] |
| Year | 0.049 | 0.016 | 3.039 | 0.026 | [0.02, 0.08] |
| Control x Year | -0.024 | 0.022 | -1.067 | 0.287 | [-0.07, 0.02] |
| Sport x Year | -0.051 | 0.022 | -2.317 | 0.021 | [-0.09, -0.01] |
