# Supplementary material for: Long-term music instruction is partially associated with the development of socioemotional skills
Source: PLoS One. 2024 Jul 18;19(7):e0307373. doi: 10.1371/journal.pone.0307373 (PMC11257369; doi:10.1371/journal.pone.0307373)
Supplement: Attachment — Submitted filename: PONE-D-23-27763 (2).docx [file pone.0307373.s004.docx]

PLOS ONE

#### Long-term music instruction partially supports the development of socioemotional skills

--Manuscript Draft--

| Manuscript Number: | PONE-D-23-27763 |
| --- | --- |
| Article Type: | Research Article |
| Full Title: | Long-term music instruction partially supports the development of socioemotional skills |
| Short Title: | Effects of music instruction on socioemotional skills |
| Corresponding Author: | Jed Villanueva  University of Southern California  Los Angeles, California UNITED STATES |
| Keywords: | El-Sistema inspired program; middle childhood; longitudinal; formal music instruction; empathy; sharing; entrainment; pitch-matching; socioemotional skills |
| Abstract: | The musical and socioemotional development of eighty-three children (age at baseline, M = 6.81) enrolled in either a music, sports, or no after-school program was followed over four years. Individuals in the music program received formal and systematic instruction in music theory, technique, and performance. Most participants in the control groups received no music education; however, in some instances, participants received minimal music education at school or at church. A pitch-matching and drumming-based rhythmic entrainment task was used to measure musical development. Sharing behavior was measured using a variation of the dictator game, and empathy was assessed using three different assessments: the Index of Empathy for Children and Adolescence, the Reading the Mind in the Eyes Test, and a Fiction Emotion-Matching task. Results revealed no time-related associations in pitch- matching ability; however, formal music instruction did improve pitch-matching relative to controls. Formal music instruction was not as effective however in improving rhythmic entrainment ability; although there were trend level differences between music and two comparison groups, the observed improvements were best explained by general age-related changes over time. This study also found limited support for a positive association between formal music instruction and socioemotional skills. That is, individuals with formal music instruction exhibited improved emotion-matching relative to those with sports training. In terms of general socioemotional development, children’s trait-level affective empathy did not improve over time, while sharing, theory of mind, and emotion-matching ability did. Finally, pitch-matching and rhythmic entrainment ability failed to reliably predict any of the socioemotional measures, with effect sizes for any association reaching trivial to small levels. |
| Order of Authors: | Jed Villanueva |
|  | Assal Habibi |
|  | Beatriz Ilari |
| Additional Information: |  |
| Question | Response |
| Financial Disclosure  Enter a financial disclosure statement that describes the sources of funding for the work included in this submission. Review the [submission guidelines](http://journals.plos.org/plosone/s/submission-guidelines#loc-financial-disclosure-statement) for detailed requirements. View published research articles from [*PLOS ONE*](http://journals.plos.org/plosone/) for specific examples.  This statement is required for submission and will appear in the published article if | The Brain and Music Program (AH) at the Brain and Creativity Institute is supported by the GRoW at Annenberg Foundation (https://growannenberg.org/), the Los Angeles Philharmonic Association [(https://www.laphil.com/),](http://www.laphil.com/)) the Van Otterloo Family Foundation, and the National Endowment for the Arts (grant 1809970-38-18, [https://www.arts.gov/).](http://www.arts.gov/)) The funders had no role in study design, data collection and analysis, decision to publish, or preparation of the manuscript. |

*Powered by Editorial Manager® and ProduXion Manager® from Aries Systems Corporation*

| the submission is accepted. Please make sure it is accurate. |  |
| --- | --- |
| Unfunded studies  Enter: *The author(s) received no specific funding for this work.*  Funded studies  Enter a statement with the following details:   - Initials of the authors who received each award - Grant numbers awarded to each author - The full name of each funder - URL of each funder website - Did the sponsors or funders play any role in the study design, data collection and analysis, decision to publish, or preparation of the manuscript? - NO - Include this sentence at the end of   your statement: *The funders had no role in study design, data collection and analysis, decision to publish, or preparation of the manuscript.*   - YES - Specify the role(s) played. |  |
| * typeset |  |
| Competing Interests  Use the instructions below to enter a competing interest statement for this submission. On behalf of all authors, disclose any [competing interests](http://journals.plos.org/plosone/s/competing-interests) that could be perceived to bias this work—acknowledging all financial support and any other relevant financial or non- financial competing interests.  This statement is required for submission and will appear in the published article if the submission is accepted. Please make sure it is accurate and that any funding sources listed in your Funding Information later in the submission form are also declared in your Financial Disclosure statement.  View published research articles from  [*PLOS ONE*](http://journals.plos.org/plosone/) for specific examples. | The authors have declared that no competing interests exist. |

*Powered by Editorial Manager® and ProduXion Manager® from Aries Systems Corporation*

| NO authors have competing interests  Enter: *The authors have declared that no competing interests exist.*  Authors with competing interests  Enter competing interest details beginning with this statement:  *I have read the journal's policy and the authors of this manuscript have the following competing interests: [insert competing interests here]* |  |
| --- | --- |
| * typeset |  |
| Ethics Statement  Enter an ethics statement for this submission. This statement is required if the study involved:   - Human participants - Human specimens or tissue - Vertebrate animals or cephalopods - Vertebrate embryos or tissues - Field research   Write "N/A" if the submission does not require an ethics statement.  General guidance is provided below. Consult the [submission guidelines](http://journals.plos.org/plosone/s/submission-guidelines#loc-guidelines-for-specific-study-types) for detailed instructions. Make sure that all information entered here is included in the Methods section of the manuscript. | The University of Southern California IRB approved the study (approval number: UP- 12-00322). Verbal assent was obtained from all child participants, and written consent obtained from the parent or guardian. |

*Powered by Editorial Manager® and ProduXion Manager® from Aries Systems Corporation*

| Format for specific study types  Human Subject Research (involving human participants and/or tissue)   - Give the name of the institutional review board or ethics committee that approved the study - Include the approval number and/or a statement indicating approval of this research - Indicate the form of consent obtained (written/oral) or the reason that consent was not obtained (e.g. the data were analyzed anonymously)   Animal Research (involving vertebrate animals, embryos or tissues)   - Provide the name of the Institutional Animal Care and Use Committee (IACUC) or other relevant ethics board that reviewed the study protocol, and indicate whether they approved this research or granted a formal waiver of ethical approval - Include an approval number if one was obtained - If the study involved *non-human primates*, add *additional details* about animal welfare and steps taken to ameliorate suffering - If anesthesia, euthanasia, or any kind of animal sacrifice is part of the study, include briefly which substances and/or methods were applied   Field Research  Include the following details if this study involves the collection of plant, animal, or other materials from a natural setting:   - Field permit number - Name of the institution or relevant body that granted permission |  |
| --- | --- |
|  |  |
| Data Availability  Authors are required to make all data underlying the findings described fully available, without restriction, and from the time of publication. PLOS allows rare exceptions to address legal and ethical concerns. See the [PLOS Data Policy](http://journals.plos.org/plosone/s/data-availability) and [FAQ](http://journals.plos.org/plosone/s/data-availability#loc-faqs-for-data-policy) for detailed information. | Yes - all data are fully available without restriction |

*Powered by Editorial Manager® and ProduXion Manager® from Aries Systems Corporation*

| A Data Availability Statement describing where the data can be found is required at submission. Your answers to this question constitute the Data Availability Statement and will be published in the article, if accepted. |  |
| --- | --- |
| Important: Stating ‘data available on request from the author’ is not sufficient. If your data are only available upon request, select ‘No’ for the first question and explain your exceptional situation in the text box. |  |
| Do the authors confirm that all data underlying the findings described in their manuscript are fully available without restriction? |  |
| Describe where the data may be found in full sentences. If you are copying our sample text, replace any instances of XXX with the appropriate details.   - If the data are held or will be held in a public repository, include URLs, accession numbers or DOIs. If this information will only be available after acceptance, indicate this by ticking the box below. For example: *All XXX files are available from the XXX database (accession number(s) XXX, XXX.*). - If the data are all contained within the manuscript and/or Supporting Information files, enter the following: *All relevant data are within the manuscript and its Supporting Information files.* - If neither of these applies but you are able to provide details of access elsewhere, with or without limitations, please do so. For example:   *Data cannot be shared publicly because of [XXX]. Data are available from the XXX Institutional Data Access / Ethics Committee (contact via XXX) for researchers who meet the criteria for access to confidential data.*  *The data underlying the results presented in the study are available from (include the name of the third party* | All data and code for analysis are available from the Open Science Framework at DOI 10.17605/OSF.IO/Y6DFC, https://osf.io/y6dfc/ |

*Powered by Editorial Manager® and ProduXion Manager® from Aries Systems Corporation*

| *and contact information or URL).*   - This text is appropriate if the data are owned by a third party and authors do not have permission to share the data.   * typeset |  |
| --- | --- |
| Additional data availability information: |  |

*Powered by Editorial Manager® and ProduXion Manager® from Aries Systems Corporation*

Manuscript	[Click here to access/download;Manuscript;Manuscript.docx](https://www.editorialmanager.com/pone/download.aspx?id=34071758&guid=e48138bc-bec6-42c6-b5c5-6998c864a2f7&scheme=1)[<https://www.editorialmanager.com/pone/download.aspx?id=34071758&guid=e48138bc-bec6-42c6-b5c5-6998c864a2f7&scheme=1>](https://www.editorialmanager.com/pone/download.aspx?id=34071758&guid=e48138bc-bec6-42c6-b5c5-6998c864a2f7&scheme=1)

1

1

2

3

##### 4 Long-term music instruction partially supports the development of socioemotional skills

5

6

7	Jed Villanueva^1^, Beatriz Ilari^2^, Assal Habibi^1*^

8

9

10

11 ^1^ Brain and Creativity Institute, Dornsife College of Letters Arts and Sciences, University of
12 Southern California, Los Angeles, CA, USA
13 ^2^ Department of Music Teaching & Learning, Thornton School of Music, University of Southern
14 California, Los Angeles, CA, USA

15

16

17

18

19 * Corresponding author
20 E-mail: [ahabibi@usc.edu](mailto:ahabibi@usc.edu) (AH)

# 21 Abstract

22 The musical and socioemotional development of eighty-three children (age at baseline, *M* =
23 6.81) enrolled in either a music, sports, or no after-school program was followed over four years.
24 Individuals in the music program received formal and systematic instruction in music theory,
25 technique, and performance. Most participants in the control groups received no music
26 education; however, in some instances, participants received minimal music education at school
27 or at church. A pitch-matching and drumming-based rhythmic entrainment task was used to
28 measure musical development. Sharing behavior was measured using a variation of the dictator
29 game, and empathy was assessed using three different assessments: the Index of Empathy for
30 Children and Adolescence, the Reading the Mind in the Eyes Test, and a Fiction Emotion-
31 Matching task. Results revealed no time-related associations in pitch-matching ability; however,
32 formal music instruction did improve pitch-matching relative to controls. Formal music
33 instruction was not as effective however in improving rhythmic entrainment ability; although
34 there were trend level differences between music and two comparison groups, the observed
35 improvements were best explained by general age-related changes over time. This study also
36 found limited support for a positive association between formal music instruction and
37 socioemotional skills. That is, individuals with formal music instruction exhibited improved
38 emotion-matching relative to those with sports training. In terms of general socioemotional
39 development, children’s trait-level affective empathy did not improve over time, while sharing,
40 theory of mind, and emotion-matching ability did. Finally, pitch-matching and rhythmic
41 entrainment ability failed to reliably predict any of the socioemotional measures, with effect
42 sizes for any association reaching trivial to small levels.

43

# 44 Introduction

45 Formal music instruction involving the intensive and systematic teaching of music
46 theory, technique, and performance, has been shown to reliably improve pitch-matching and
47 rhythmic entrainment [1-5], two fundamental abilities that shape our experiences with music.
48 Pitch-matching, or the ability to mimic the pitch of an external stimulus, requires the complex
49 coordination of vocal, motor, and auditory processes; individuals must perceive pitch and
50 temporal information, map this onto motor plans, and continuously regulate their vocal output
51 [6]. Pitch-matching emerges naturally over time [7], with general improvements occurring
52 throughout childhood [8, 9]. Musical engagement, including pitch-matching, performing,
53 listening, and dancing, is predicated on the emergence of rhythmic entrainment, or the
54 spontaneous synchronization of bodily behaviors with external rhythmic processes [10, 11]. As
55 with pitch-matching, the foundations for rhythmic entrainment emerge early in childhood [12]
56 and continue to develop with age [13, 14] and improved motor control [15]. Rhythmic
57 entrainment facilitates the coordination of bodies and voices to the musical beat (i.e., a
58 temporally structured event) through interaction. Thus, scholars often speak of temporal and
59 social aspects of entrainment [16]. Given that collective music making requires humans to
60 synchronize their bodies and voices to the musical beat, formal music instruction is expected to
61 further augment rhythmic entrainment by nurturing one’s ability to detect temporal patterns
62 within complex stimuli (i.e., music) and form predictions accordingly. In terms of musical
63 engagement, rhythmic entrainment enables individuals to move in synchrony to the beat of a
64 song and monitor multiple musical events within an auditory scene [17]. This phenomenon can
65 then be observed through physical behaviors such as tapping, head bobbing, and dancing. When
66 applied to social interactions, rhythmic entrainment allows individuals to better synchronize
67 motor and vocal behaviors with another person [18-20].
68 Beyond their role in music perception, pitch-matching and rhythmic entrainment are vital
69 in shaping our social interactions. For instance, pitch-matching is implicated with establishing
70 social bonds and enabling effective communication through vocal synchrony and emotion
71 recognition [21]. On the other hand, rhythmic entrainment offers a more direct relationship with
72 social outcomes; the temporal synchronization of physical behaviors with others improves
73 cooperation [22-24], social bonding [26, 27], and the development of a collective identity [28].
74 Vocal synchrony is the phenomenon by which individuals modulate their speaking
75 patterns to align with their conversation partners. This includes adjustments in pronunciation
76 [29] and syntactic structure [see 30], as well as the modulation of prosodic cues such as speaking
77 rate [31], voice intensity [32], and pitch [33, 34]. Such adaptation in speech encourages
78 interlocutors to adopt the same mental representations, decreasing misunderstandings and
79 improving perceptions of familiarity [33], attractiveness and likability [35, 36], and rapport [37].
80 For instance, high levels of pitch accommodation during combative conversations (i.e., problem-
81 solving during couples therapy) has been correlated with positive rather than negative attitudes
82 [38]. Similarly, in a study evaluating the performance of therapists during psychological
83 counseling, Imel et al. [34] identified sessions with high levels of empathy to correlate with a
84 greater degrees of vocal pitch synchronization between the therapist and patient. Altogether, this
85 mirroring of vocal behaviors improves successful communication and leads to positive social
86 perceptions of conversation partners. However, the ability to accurately identify prosodic cues
87 varies greatly among individuals, causing widespread interest in determining these contributing
88 factors.
89 Given evidence that formal music instruction enhances auditory and linguistic processing
90 [see 39], considerable attention has been devoted to evaluating the musician’s advantage for
91 prosodic perception. In fact, musicians demonstrate improved perception for prosodic cues such
92 as pitch [40-42] rhythm/timing [43-45], and timbre [46]. Consistent with these findings,
93 musicians generally outperform nonmusicians in identifying emotions in both musical and non-
94 musical stimuli (e.g., [47-50], but see [51]). Notably, a recent study by Greenspon and
95 Montanaro [21] found a significant correlation between pitch-matching and vocal emotion
96 recognition, suggesting that individuals with better pitch-matching ability may be better
97 equipped at understanding the emotions in another’s voice. This enhanced auditory processing
98 might then explain why musicians appear to exhibit stronger and more consistent affective
99 responses to musical stimuli (e.g., [52-54]).
100 As for rhythmic entrainment, the effects of interpersonal synchrony on socioemotional
101 behavior are extensive and can be explained through the blurring of self-other boundaries, and an
102 increased allocation of attentional resources towards group members (see [55]). As such,
103 individuals interacting in time have been found to improve on multiple dimensions of prosocial
104 behavior ([24], [26], [56]), or voluntary actions intended to benefit another individual [25]. For
105 example, Kirschner and Tomasello [23] found that 4-year-old children were more likely to
106 spontaneously help and cooperate with another child after playing children’s musical games that
107 involve singing and dancing. Likewise, 8-year-old children engaged in a group singing activity
108 were more likely to cooperate during a prisoner’s dilemma game, than children involved in a
109 group art or competitive games activity [22]. However, it seems that engaging in the same
110 behavior may be insufficient in explaining these socioemotional gains. In a study by Hove and
111 Risen [26], participants completed a task which involved tapping with an experimenter. Once
112 completed, participants were asked to rate the experimenter’s likeability. Hove and Risen found
113 that the degree of tapping synchrony between participant and experimenter predicted affiliation
114 ratings, and not just the shared act of tapping together. Similar results were also found in
115 developmental studies, whereby, children’s social attitudes improved more when tapping in
116 synchrony with a partner than simply tapping together [57]. Yet this prosocial effect of
117 synchronization accuracy was not found when evaluating spontaneous helping and sharing
118 behaviors [58].
119 Beyond the near transfer of music-related skills, formal music instruction has been
120 associated with the development of more general aspects of cognition such as speech-in-noise
121 recognition [59], executive functioning [60], language [61], and visuospatial skills [62]. It stands
122 to reason that improvements in pitch-matching ability and rhythmic entrainment would also
123 result in an increase in socioemotional skills given their associations with empathic responding
124 and social cohesion.
125 In general, large age-related changes in socioemotional skills can be observed early
126 during infancy. This transition between infancy and toddlerhood sees a notable increase in
127 related capacities such as social-cognitive awareness, empathic responding, and self-other
128 distinction. For instance, rudimentary forms of prosocial behavior and expectations for helping
129 and cooperation have been observed in 9- to 18-month-olds [63]. As babies transition into early
130 childhood (e.g., ages 3 and 4), their comprehension of social situations begins to incorporate
131 more sophisticated elements such as an understanding that other individuals possess their own
132 thoughts, beliefs, and emotions or theory of mind [64]. Similarly, middle childhood (e.g., 5- to
133 10-years-old) has been shown to coincide with substantial gains in the empathy domain, such as
134 the development of an advanced theory of mind, or an understanding that other individuals may
135 hold inaccurate beliefs about the beliefs of a third party [65].
136 In research on the neural basis of social behavior, adolescents compared to adults have
137 exhibited decreased functional activity in the medial prefrontal cortex (mPFC; [66-68]) and
138 increased activity in the temporoparietal junction (TPJ; [69]) during social tasks. Findings from
139 adult (e.g., [70-73]) and lesion studies (mPFC, [74, 75]; TPJ, [76]) generally demonstrate a
140 reliance on these areas to perform theory of mind tasks. Likewise, in their longitudinal analysis
141 of 7- to 30-year-olds, Mills et al. [77] found gray matter volume and cortical thickness in the
142 mPFC and TPJ to decrease from childhood into adulthood. Similarly, the surface area for these
143 regions peaked in early/pre-adolescence (i.e., 8- to 11-years-old) before decreasing during
144 adulthood. Therefore, the functional changes and anatomical development of these areas might
145 reflect adaptations into how children understand the mental states of others.
146 Research into middle childhood is of particular interest, as it represents a period during
147 which socioemotional abilities are greatly influenced by developments in self-awareness and
148 adaptations to new environmental pressures [78]. In a longitudinal study measuring the
149 development of cooperative, helping, and sharing behaviors in 6- to 12-year-olds, Malti et al.
150 [79] found each of these prosocial behaviors to follow unique trajectories. Cooperation improved
151 over time; however, rather than identifying an anticipated improvement in helping behavior,
152 children actually decreased in their willingness to do so. Likewise, sharing followed a nonlinear
153 inverted U-shaped trend, with peak sharing occurring at around 9 years of age.
154 In the music cognition literature, the exploration of socioemotional skills is obscured by
155 inconsistent operationalizations of core constructs (e.g., empathy, [80]; as cited by [81]),
156 differences on music education approaches and contextual factors. For instance, joint music
157 making has been found to both increase [22] and have no effect [58] on sharing behaviors. A
158 similarly nuanced phenomena was found among preschoolers, whereby engaging in musical play
159 increased spontaneous helping and overall sharing, but not spontaneous sharing [82]. In this
160 paper, we refer to socioemotional skills as traits and capacities which contribute to an
161 individual’s ability to maintain social relationships and respond to the emotions of others.
162 Socioemotional skills encompass multiple psychological constructs important for impacting an
163 individuals’ overall well-being. Self-esteem, empathy, prosociality, and theory of mind all fit
164 into this umbrella term as they are integral in assisting individuals engage in positive social
165 interactions. Within this larger framework, formal music instruction may enhance the
166 development of some of these skills. In a study by Rickard et al. [83], school-aged children
167 received either Kodály music classes or group string instrument training in a standard classroom
168 environment. They found that music classes helped combat a decline in self-esteem experienced
169 by those in the control condition. Similarly, Rabinowitch, Cross, and Burnard [84] found that
170 engagement in a 9-month music program, focused on playing musical games specialized in
171 encouraging empathy-inducing behaviors (e.g., entrainment, imitation, shared intentionality),
172 improved trait empathy in school-aged children. Conversely, Costa-Giomi [85] randomly
173 assigned children into two groups – one which received individual piano lessons and the other no
174 formal music instruction. The self-esteem of children in the experimental condition increased
175 significantly over three years; however, at no point did their scores differ from the control group.
176 Finally, in a large-scale investigation of school-based musical instruction, Rickard, Bambrick,
177 and Gill [86] found no significant improvements on a comprehensive battery of psychosocial
178 well-being measures.
179 Importantly, research that fails to identify a relationship between formal music instruction
180 and socioemotional development often consist of one-on-one music instruction (e.g., [85], [87-
181 89]). By contrast, research on the involvement of group-based musical activities generally
182 supports a positive association with socioemotional development or some of its components
183 (e.g., [57], [84], [90-93]), although research revealing few differences between music students
184 and other groups (e.g., dancers) also exist [94]. Finally, it is also important to account for the fact
185 that research on formal music instruction tends to be based on cross-sectional or short-term
186 longitudinal designs, which may fail to capture the developmental changes of a long-term
187 commitment such as music.
188 Using data collected from a 7-year longitudinal study, we aimed to understand the
189 development of pitch-matching, rhythmic entrainment, and socioemotional skills in children who
190 received formal music instruction and other non-music based after-school programs. In addition,
191 we aimed to explore the social implications of pitch-matching and replicate the positive
192 relationship between rhythmic entrainment and socioemotional skills.

# 193 Methods

194 Data and code for analysis are available at <https://osf.io/y6dfc/>.

## 195 Participants

196 Eighty-three children (ages 5.73–8.01 years old at baseline, *M* = 6.81 years, *SD* = 0.54
197 years; 59% male, 41% female) were recruited from community and enrichment programs of the
198 greater Los Angeles area to participate in a 7-year longitudinal study. All children self-selected
199 into their program and were raised in bilingual communities but attended English speaking
200 schools. Research protocols were approved by the University of Southern California Institutional
201 Review Board (UP-12-00322). Verbal assent was obtained from all child participants, and
202 informed written consent was obtained from the parent or guardian in their preferred language.
203 Children received toys and small prizes while parents received monetary compensation for their
204 child’s participation. Data collection occurred between September 17^th^, 2012, and March 4^th^,
205 2020. Subsequently, data was accessed on September 21^st^, 2022 and analyzed anonymously.
206 Given the longitudinal nature of this study, not all participants could be evaluated with
207 every measure at every time point (see S1 Table). To evaluate the relationship between gender
208 and an incomplete set of observations, a Chi-Square Test of Independence was performed
209 indicating that there is no relationship between gender and missing data (χ^2^1 < 0.001, p = 1).
210 Likewise, Wilcoxon Rank Sum Tests verified that individuals with missing data did not differ in
211 age, cognitive ability, or any of the dependent variables analyzed in this study.

### 212 After-school programs

213 All after-school programs were free and intended for families from underserved
214 communities in Los Angeles. Two cohorts of approximately age-matched participants were
215 enrolled in either a music (*n* = 26) or sports (*n* = 28) program. A similarly matched control group
216 (*n* = 29) was recruited from public schools in Los Angeles, consisting of children uninvolved
217 with any systematic after-school program at the time of recruitment. We continued to monitor
218 this group’s participation in after-school programs to ensure they did not engage in any intensive,
219 formal music instruction program over the course of the study.
220 Participants in the music group were enrolled in the Youth Orchestra of Los Angeles at
221 the Heart of Los Angeles. This intensive music program followed the Venezuelan “El Sistema”
222 approach towards music education, which emphasizes a communal approach to music making
223 via sectional, ensemble, and orchestral practices. Children in this program met four to five times
224 a week for 6 to 7 hours, during which, they learned about the fundamentals of music, engaged in
225 group lessons, and occasionally performed recitals.
226 Participants in the sports group were enrolled in either a community-based swimming or
227 a soccer program. Beyond the development of sport-related skills, both programs taught children
228 the importance of health, nutrition, and sportsmanship. As with formal music instruction, sports
229 are demanding sensorimotor activities that require long-term attentive practice to develop.
230 Importantly, participants in the sports group did not engage in any systematic or long-term
231 formal music instruction as verified by systematic annual interviews with their families. Children
232 in the swimming program attended two one-hour training sessions each week and attended
233 competitions on the weekend. Those in the soccer program attended three two-hour sessions
234 every week, in addition to a one-hour game on the weekend.

## 235 Measures

### 236 Pitch-matching task

237 Pitch-matching was measured through a singing task, during which participants repeated
238 back 10 melodies sung by an experimenter (Fig 1). Participant and experimenter audio were
239 recorded as either an MP3 or WAV file, and then converted to the frequency-domain using a
240 Discrete Fourier Transform available in Melodyne. In cases where participant responses were
241 affected by vocal fry, the calculated Hertz value would be an octave lower than audibly
242 perceived. These values were corrected by doubling the frequency of the pitch as to raise the
243 pitch by one octave.
244 Pitch-matching values were calculated in a similar manner to Pfordresher and Brown [95]
245 by comparing the Hz of a target and response pitch. For our study, the target pitch was the audio
246 sung by the experimenter, while the response pitch was that sung by the participant. For each of
247 the 10 melodies, the difference between the experimenter and participant’s pitches were
248 calculated to represent an individual pitch deviation score. These scores were first averaged, then
249 subtracted from 100, then divided by 100 to create a normalized singing score. In the case that
250 combined individual pitch deviation scores were greater than 100Hz, indicating roughly twice
251 the deviation from the target pitch, the sample was scored as a zero. The 10 normalized scores
252 were then averaged to create an overall pitch-matching score ranging from 0 (speech/chant-like
253 or a difference greater than two steps from the target pitch) to 1 (no difference in frequency
254 between the sung pitch and the target pitch).
255 Participants at baseline were only asked to repeat sections 1-3 and 7-8, which came from
256 component 6 of the AIRS Test Battery of Singing Skills [96]. Melodies 4-6 and 9-10 were
257 developed by Ilari, Fesjian, and Habibi [97] to supplement those from year 1 onward.
258 **Fig 1. Pitch-matching task stimuli.** Musical score showing the melodies sung during the task.
259 Each numbered section represents a melody the participant first heard then echoed back to the
260 experimenter. Sections 1-3, 4-6, 7-8, and 9-10 were grouped into four musical phrases during
261 analysis.

### 262 Rhythmic entrainment

263 Rhythmic entrainment was measured following Kirschner and Tomasello [98]. Children
264 were told to drum at different tempi with either a pre-recorded beat, or together with the pre-
265 recorded beat and an experimenter [see 97]. These “alone” and “social” conditions were
266 counterbalanced during presentation to prevent ordering effects. During the task, participants sat
267 across from the experimenter with a drum placed in front of them. Stimuli would play from a
268 laptop through a guitar amplifier which participants would then drum along to. A microphone
269 inside the drum recorded the audio for each participant’s response. This response audio was then
270 compared to the audio of the original stimulus using a custom MATLAB [99] script applying
271 circular statistics to measure their synchronization accuracy along a moving time window [see
272 98]. Values ranged from 0-1, with a value of 0 representing low synchronization accuracy and 1
273 representing perfect synchronization. Rhythmic stimuli included isochronous 120- and 180-bpm
274 beats. The 120-bpm condition was administered each year; however, the 180-bpm was not
275 administered in year 1 of the study.

### 276 Sharing task

277 From years 2 to 4 of the study, sharing was measured through a variation of the dictator
278 game [100]. In year 2, children were asked to choose 10 stickers as rewards for completing
279 different tasks throughout their behavioral assessment. Afterwards, they were given the option to
280 share their stickers with a pictured stranger of the same age and gender. Participants were
281 explicitly told that sharing would result in no benefit to themselves, and that no one would know
282 how many stickers, if any, they shared. After receiving these instructions, children were left
283 alone for 2-3 minutes to place however many stickers they wanted to share in a separate bag.
284 Once complete, the number of stickers was counted by an experimenter while another researcher
285 distracted them in a separate room. In years 3 and 4 of the study, the sharing task was modified
286 to include 15 total stickers, as well as an additional option for children to share with a friend of
287 similar age and no familial relation. To make the sharing task comparable across time, the total
288 proportion of stickers given away was used as a measure of sharing.

### 289 Empathic responding and theory of mind

290 From baseline to year 4, children completed the Index of Empathy for Children and
291 Adolescents (IECA; [101]), the Reading the Mind in the Eyes Test of theory of mind (Eyes Test;
292 [102]), and a modified variation of the Fiction Emotion-Matching task [103]. The IECA is a 22-
293 item questionnaire frequently used to assess the dispositional affective empathy of children and
294 adolescents. Participants are prompted with phrases such as “People who kiss and hug in public
295 are silly.”, or “Some songs make me so sad I feel like crying.”, which they must respond yes or
296 no to. The Eyes Test is a ubiquitous measure for theory of mind, during which participants view
297 28 black-and-white photos of an adult’s eyes. Children are then asked to evaluate and select one
298 of four options representing the emotional state of the pictured individual.
299 During the Fiction Emotion-Matching task, participants viewed a set of fictional movie
300 clips exemplifying a target emotion (i.e., happiness, sadness, fear) and then evaluated the
301 emotional state of the character. After completion of the clip, they were asked to explain what
302 emotion the character was feeling, their own emotional state, and the degree to which they felt
303 sympathy towards the character. Participants were scored based on the congruency of the
304 character’s identified emotion and their own emotion. Scores were either 0 (incongruent) or 1
305 (congruent) for each clip, which were then averaged together as a holistic evaluation of cognitive
306 and affective empathy. At baseline, participants viewed four 30-second clips from *Charlie & the*
307 *Chocolate Factory* (see [103]). From years 1 through 4, more age-appropriate clips were selected
308 from *The Land Before Time* (sad), *My Girl* (sad), *Jumanji* (fear), *Little Miss Sunshine* (happy),
309 *American Girl* (sad), and *The Sandlot* (fear). Goldstein and Winner originally scored wholly
310 exact matches as correct; however, we also opted to score conceptually similar emotions as
311 correct if both emotions were generally aligned in terms of valence and arousal. For example,
312 answering “excited” in response to the clip from *Little Miss Sunshine* (happy) would result in a
313 correct match.

### 314 Cognitive ability

315 To measure general cognitive ability, each participant completed the Block Design,
316 Matrix Reasoning, Vocabulary, and Similarities subtests of the Wechsler Abbreviated Scale of
317 Intelligence (WASI-II; [104]). The Block Design and Matrix Reasoning tasks are well validated
318 measures for problem solving and visuospatial skills. Likewise, the vocabulary and similarities
319 subtests are measures of general knowledge or crystallized intelligence. In the Block Design
320 task, participants saw and were asked to recreate a pictured design using a set of blocks. In the
321 Matrix Reasoning task, participants were shown a group of images and asked to select which
322 pattern, from a set of four or six options, completes the image. The Vocabulary Test had children
323 orally define different words, and the Similarities Test had children orally describe the
324 similarities between two common objects or concepts. A Full-Scale Intelligence Quotient (FSIQ-
325 4) score was then calculated as a composite measure of all four subtests.
326 Participants also completed the Digit-Span task as a measure of auditory short-term
327 memory (forward condition) and working memory (backwards condition). The experimenter
328 read-aloud a sequence of numbers for the children to repeat back either in the order they heard it
329 (forwards) or in reverse (backwards) order. As participants responded correctly, the number of
330 digits in a sequence increased until they responded incorrectly to two trials in a row.

## 331 Procedure

332 Children were tested individually on a large battery of cognitive, socioemotional, and
333 motor tasks each year at either the program site or at the Brain and Creativity Institute at the
334 University of Southern California, over several days. During each testing session, participants
335 completed the music-production-related tasks, followed by the empathy measures, and finally the
336 sharing task. Testing could occur over multiple sessions depending on participant availability.

# 337 Results

## 338 Data analysis

339 Analysis was conducted in R [105] and processed using the tidyverse [106]. Linear mixed
340 effects models were built using the lme4 package [107], with p-values generated by lmerTest
341 [108]. For issues in convergence, the afex package [109] was used to identify optimizers. Due to
342 disruptions caused by the COVID-19 pandemic, data collection was interrupted during the fifth
343 year and was resumed the following year. As such, analysis only includes data from baseline to
344 year 4. An alpha value of *p* < .05 was used to determine significance for all analyses.
345 Correlations between measures of empathy and sharing were calculated as an estimation
346 for construct validity within the socioemotional domain. Some but not all correlations were
347 significant, with the few significant Pearson’s *r* ranging from 0.21 (Eyes Test vs. IECA) to 0.24
348 (Fiction Emotion-Matching vs. IECA), suggesting that each measure assessed a separate
349 dimension of social cognition (see Table 1).

##### 350 Table 1. Correlation Matrix of Socioemotional Measures.

| Variables | Stickers Away | IECA | Eyes Test | Emotion-Matching |
| --- | --- | --- | --- | --- |
| Stickers Away  IECA | —  0.23** | — |  |  |
| Eyes Test | 0.14 | 0.21*** | — |  |
| Emotion-Matching | 0.06 | 0.24*** | 0.07 | — |

351 Matrix depicting the Pearson’s correlation coefficients between each socioemotional measure.
352 Principal component analysis (PCA) was used to reduce the dimensionality and
353 multicollinearity of our regression analysis by creating a single composite score from measures
354 intended to assess the same construct. The resulting outcome variables were the product of a
355 PCA conducted on all the available data at every time point. This allowed us to best capture
356 time-related changes and maximize comparability between years.
357 For the pitch-matching task, Pearson’s *r* ranged from 0.68 (phrases 2 and 3) and 0.88
358 (phrases 1 and 2). The resulting component representing “pitch-matching” was extracted from
359 the scores for each musical phrase. Although the pitch-matching task was administered each
360 year, the full task involving all four musical phrases was not administered until year 1 of the
361 study. Values for pitch-matching at baseline were not generated for this year given the
362 incomplete data. The subsequent component explained 82.5% of the variance, with factor
363 loadings ranging from 0.84 to 0.94.
364 The component “rhythmic entrainment” was extracted from participant’s synchronization
365 accuracy to the 120- and 180-bpm stimuli in the alone and social conditions; however, data from
366 the 180-bpm condition were not collected during Year 1. Consequently, as is the case with pitch-
367 matching baseline, rhythmic entrainment values were not generated for year 1. Unlike the pitch-
368 matching task, the range of correlations between the measures were markedly lower (120-bpm
369 alone vs. 120-bpm social, *r* = 0.36; 180-bpm alone vs. 180-bpm social, *r* = 0.68). The resulting
370 principal component explained 64% of the variance, with factor loadings ranging from 0.72 to
371 0.88. A PCA was not conducted on the socioemotional or cognitive measures due to their weak
372 intercorrelations, in addition to better maintaining interpretability for each outcome variable.

## 373 Growth curves

374 Linear mixed effects models were created to measure the growth of rhythmic
375 entrainment, pitch-matching, sharing, and empathy over time, and to determine if any of these
376 variables varied as a function of formal music instruction. Year, group (e.g., music, sports, and
377 control), and their interaction were included as fixed effects in all models to evaluate these
378 questions. FSIQ-4 and working memory were tested as covariates in all four models. In addition,
379 auditory short-term memory was included as a covariate for the pitch-matching and rhythmic
380 entrainment models. Gender was included as a covariate for the socioemotional models since
381 females have been shown to perform better at empathy-related tasks [101, 110]; however, later
382 analysis would reveal that FSIQ-4 and working memory were the only preferred covariates.
383 Model selection was performed by choosing the model with the lowest Bayesian information
384 criteria in order to penalize models with increased complexity. All models had a random effects
385 structure including by-participant random intercepts and slopes for time to account for individual
386 differences in rates of change. The music group was set as the reference level from which all
387 comparisons were made.

### 388 Music Production

389 FSIQ-4 score was a significant predictor for pitch-matching, whereby every unit increase
390 in FSIQ-4 improved pitch-matching by an average of 0.017 points (*β* = 0.006, *SE* = 0.07, *t* =
391 2.603, *p* = .01; *d* = 0.20, 95% CI [0.05, 0.35]; see S2 Table for model estimates). In addition,
392 pitch-matching over time decreased significantly for individuals in the control condition relative
393 to the music condition, with an average decrease in rate by 0.26 points (*β* = -0.26, *SE* = 0.10, *t* = -
394 2.51, *p* = 0.016, see Fig 2). Year failed to reach significance (*β* = 0.6, *SE* = 0.07, *t* = 0.80, *p* =
395 0.43; *d* = 0.24, 95% CI [-0.03, 0.51], indicating no natural changes due to development alone
396 over time. Since baseline PCA scores were not calculated, pitch-matching at this timepoint was
397 evaluated by taking the average of the available data (phrases 1 and 3). This averaged value was
398 then used as the outcome variable in a linear model predicted by group. Model estimates indicate
399 that individuals in the music group scored about 0.13 points higher than those in the sports group
400 (*β* = 0.13, *SE* = 0.06, *t* = 2.15, *p* = 0.036; see Fig 3).
401 **Fig 2. Pitch-matching growth curve.** Pitch-matching score plotted against time. Only
402 individuals in the music group improved on pitch-matching ability over time. Grey shading
403 indicates the 95% confidence interval for the lines of best fit.
404 **Fig 3. Baseline pitch-matching and rhythmic entrainment scores.** Violin plot depicting pitch-
405 matching and rhythmic entrainment scores at baseline. (A) Pitch-matching scores were
406 calculated as the average pitch-matching score of phrases 1 and 3 (Music, *M* = 0.67 *SD* = 0.24;
407 Control, *M* = 0.59, *SD* = 0.17; Sport, *M* = 0.54, *SD* = 0.20). (B) Baseline rhythmic entrainment
408 scores were generated by conducting a PCA on all available rhythmic entrainment data (Music,
409 *M* = -0.33 *SD* = 0.91; Control, *M* = -0.95, *SD* = 0.83; Sport, *M* = -0.64, *SD* = 1.25). Colored dots
410 visualize individual observations with black dots indicating group averages.
411 Rhythmic entrainment scores did not differ at baseline; however, individuals in the
412 control group did trend towards scoring 0.62 points less than the music group (*β* = -0.62, *SE* =
413 0.32, *t* = -1.95, *p* = 0.056; see Fig 3). Model estimates reveal that rhythmic entrainment is
414 significantly predicted by year (*β* = 0.29, *SE* = 0.04, *t* = 6.84, *p* < .001; *d* = 1.11, 95% CI [0.87,
415 1.34]; see Fig 4), indicating a strong natural development in the ability over time. Although the
416 model did not reveal any group differences in rates of change, when year is held constant, the
417 control group scored about 0.70 points lower (*β* = -0.70, *SE* = 0.29, *t* = -2.54, *p* = .013) than the
418 music group on the rhythmic entrainment task. Similarly, the sports group trended towards
419 scoring about 0.51 points less (*β* = -0.51, *SE* = 0.29, *t* = -1.79, *p* = 0.08) than the music group.
420 **Fig 4. Rhythmic entrainment growth curve***.* Rhythmic entrainment score plotted against time.
421 There is a strong linear trend for all groups in relation to year; however, no significant
422 differences in the rate of change between groups. Grey shading indicates the 95% confidence
423 interval for the lines of best fit.

### 424 Socioemotional skills

425 A significant effect of time on sharing was found, with an average increase of 7.2%

426	stickers given away each year (*β* = 0.072, *SE* = 0.031, *t* = -2.28, *p* = .024; *d* = 0.53, 95% CI

427 [0.36, 0.70]). A significant group by year interaction was also found, whereby individuals in the
428 sports conditions gave away stickers about 9% more than the music group each year (*β* = 0.09,
429 *SE* = 0.04, *t* = 2.054, *p* = .042). Additional analyses found the overall shape of the sharing
430 distributions to follow an inverted U-shape trend over time (see Fig 5), peaking at year 3 (*M* =
431 0.62, *SD* = 0.16).
432 **Fig 5. Distribution of stickers given away.** Violin plot depicting the proportion of stickers
433 given away to either a stranger or a friend. The proportion of stickers shared followed an
434 inverted U-shaped trend, with an average of 32% of stickers (*SD* = 0.18) given away at year 2,
435 62% (*SD* = 0.16) at year 3, and 54% (*SD* = 0.20) at year 4. Colored dots represent individual
436 participants and their respective group.
437 Measures of empathy did not show a uniform pattern of results with respects to group,
438 year, or cognitive abilities, highlighting their lack of convergent validity (see Fig 6; see S3 Table
439 for model estimates). For instance, the IECA’s had a main effect of working memory (*β* = 0.018,
440 *SE* = 0.006, *t* = 2.91, *p* = .004; *d* = 0.17, 95% CI [0.05, 0.28]) while the Eyes Test varied
441 significantly as a function of only time (*β* = 0.05, *SE* = 0.008, *t* = 5.94, *p* < .001; *d* = 1.37, 95%
442 CI [1.05, 1.68]). However, the Fiction Emotion-Matching task found time (*β* = 0.05, *SE* = 0.02, *t*

443	= 3.04, *p* = .003; *d* = 0.16, 95% CI [0.04, 0.28]) and the sport by year interaction to be

444 significant, whereby the sports group performed -0.05 points worse than the music group each
445 year (β = -0.05, SE = 0.02, t = -2.32, p = .021).
446 **Fig 6. Socioemotional skills growth curves.** Measures of socioemotional skill plotted against
447 time. Performance on the (A) sharing, (B) IECA and (C) Eyes Test improved over time, while
448 performance on the (D) Fiction Emotion-Matching task did not. No differences in rate of change
449 were found in any measure apart from the Emotion-Matching task, where individuals in the
450 sports group performed on average 0.05 points worse than the music group each year (*p* =
451 0.021). Grey shading indicates the 95% confidence interval for the lines of best fit.

## 452 Music production and socioemotional skills

453 A second set of analyses was conducted predicting socioemotional skills as a function of
454 music production (i.e., pitch-matching or rhythmic entrainment). Year and by-participant random
455 slopes for time were again included in all models as fixed and random effects, respectively.
456 FSIQ-4 and working memory were added as covariates and evaluated by their model’s respective
457 BIC.
458 As shown in Table 2, each unit increase in pitch-matching ability decreased the
459 proportion of stickers shared by about 3.5% (*β* = -0.035, *SE* = 0.01, *t* = -2.38, *p* = .019; *d* = 0.19,
460 95% CI [0.03, 0.34]). As with the analysis of pitch-matching’s growth curve, time continues to
461 be a robust and significant predictor in the model. On the other hand, rhythmic entrainment
462 failed to reach significance as a predictor in the sharing model.

##### 463 Table 2. Music Production Scores Predicting Sharing with a Stranger.

Effect	*β	SE	t	p	95% CI*

Sharing ~ pitch-matching

| Intercept | 0.121 | 0.058 | 2.119 | 0.036 | [0.01, 0.24] |
| --- | --- | --- | --- | --- | --- |
| pitch-matching | -0.035 | 0.015 | -2.379 | 0.019 | [-0.06, -0.01] |
| Year | 0.128 | 0.019 | 6.752 | < 0.001 | [0.09, 0.17] |

Sharing ~ rhythmic entrainment

Intercept	0.140	0.059	2.368	0.020	[0.02, 0.26]

entrainment

| rhythmic -0.002 | 0.019 | -0.148 | 0.882 | [-0.04, 0.03] |
| --- | --- | --- | --- | --- |
| Year 0.120 | 0.020 | 5.922 | < 0.001 | [0.08, 0.16] |

464 CI = confidence interval; Regression model estimates predicting proportion of stickers given
465 away as a function of either pitch-matching or rhythmic entrainment ability. Sharing was found
466 to vary negatively with respects to pitch-matching, while no significant relationship with
467 rhythmic entrainment was found. In general, pitch-matching ability reduced the proportion of
468 stickers given away by about 3.5%. Year was again a significant predictor in both models.
469 Few consistent patterns were found between the music production measures and
470 empathy; one of which being the failure for pitch-matching to predict any empathy measure
471 (Table 3). Rhythmic entrainment displayed a trending relationship in both the IECA (p = 0.07, d

472	= 0.16, 95% CI [-0.02, 0.34]) and the Eyes Tests (p = 0.08, d = 0.12, 95% CI [-0.01, 0.26]), but

473 failed to reach significance in the emotion-matching model (see Table 4).

##### 474 Table 3. Pitch-Matching Predicting Empathy Measures.

Effect	*β	SE	t	p	95% CI*

IECA ~ pitch-matching

| Intercept | 0.425 | 0.035 | 12.423 | < 0.001 | [0.37, 0.50] |
| --- | --- | --- | --- | --- | --- |
| pitch-matching | -0.002 | 0.010 | -0.161 | 0.873 | [-0.02, 0.02] |
| Year | 0.021 | 0.008 | 2.485 | 0.016 | [0.00, 0.04] |
| working memory | 0.018 | 0.007 | 2.542 | 0.012 | [0.00, 0.03] |
| Eyes Test ~ pitch-matching | | | | | |
| Intercept | 0.576 | 0.018 | 32.811 | < 0.001 | [0.54, 0.61] |
| pitch-matching | 0.002 | 0.008 | 0.245 | 0.806 | [-0.01, 0.02] |
| Year | 0.035 | 0.006 | 6.258 | < 0.001 | [0.02, 0.05] |
| Fiction Emotion-Matching ~ pitch-matching | | | | | |
| Intercept | 0.772 | 0.043 | 18.162 | < 0.001 | [0.69, 0.86] |
| pitch-matching | 0.004 | 0.017 | 0.218 | 0.827 | [-0.03, 0.04] |
| Year | 0.014 | 0.013 | 1.085 | 0.280 | [-0.01, 0.04] |

475 CI = confidence interval; Regression model estimates predicting empathy measures as a function
476 of pitch-matching. Pitch-matching failed to predict any empathy measures at both a significant
477 and trending level. The driving predictor for most tasks was instead time, which improved
478 performance on IECA by about 0.02 points (*p* = 0.016) and the Eyes Test by about 0.03 points (*p*
479 < .001) each year. For each unit increase in working memory, IECA score also improved by
480 about 0.02 points (*p* = 0.012). The Fiction Emotion-Matching task failed to vary as a function of
481 either predictor.

##### 482 Table 4. Rhythmic Entrainment Predicting Empathy Measures.

Effect	*β	SE	t	p	95% CI*

IECA ~ rhythmic entrainment

Intercept	0.537	0.018	29.477	< 0.001	[0.50, 0.57]

rhythmic entrainment

0.019	0.011	1.805	0.074	[-0.00, 0.04]

Year	0.013	0.007	1.726	0.088	[-0.00, 0.03]

Eyes Test ~ rhythmic entrainment

Intercept	0.517	0.019	27.001	< 0.001	[0.48, 0.56]

rhythmic entrainment

0.018	0.010	1.764	0.079	[-0.00, 0.04]

Year	0.052	0.006	9.006	< 0.001	[0.04, 0.06]

Fiction Emotion-Matching ~ rhythmic entrainment

Intercept	0.684	0.043	16.022	< 0.001	[0.60, 0.77]

rhythmic entrainment

0.035	0.025	1.431	0.154	[-0.01, 0.08]

Year	0.032	0.014	2.298	0.024	[0.00, 0.06]

483 CI = confidence interval; Regression model estimates predicting empathy as a function of
484 rhythmic entrainment. Rhythmic entrainment failed to reach significance for all empathy
485 measures; however, was trending towards significance for both the IECA (*p* = 0.07) and Eyes
486 Tests (*p* = 0.07). Year was the only significant predictor in any of the models, with an average
487 increase of about 0.05 points in the Eyes Test (*p* < .001) and 0.03 points on the Fiction Emotion-
488 Matching task (*p* = 0.024) over time. Year was trending towards significance (*p* = 0.09) in the
489 IECA model.
490 Instead of finding an effect of music production on socioemotional outcomes, an effect of
491 time was found for most models. Interestingly, the effect of year on IECA and Fiction Emotion-
492 Matching performance differed depending on which music production measure was included as a
493 fixed effect; IECA score was significantly predicted by year (*p* = 0.016, *d* = 0.34, 95% CI [0.08,
494 0.60]) only when pitch-matching was included in the model. Likewise, Fiction Emotion-
495 Matching score was only significant when rhythmic entrainment was included in the model (*p* =
496 0.024, *d* = 0.15, 95% CI [-0.04, 0.25]).
497 The only empathy measure to consistently improve over time was the Eyes Test, which
498 increased each year by an average of 0.035 points (*β* = 0.035, *SE* = 0.006, *t* = 6.26, *p* < .001; *d* =
499 0.81, 95% CI [0.52, 1.10]) or 0.052 points (*β* = 0.052, *SE* = 0.006, *t* = 9.01, *p* < .001; *d* = 0.92,
500 95% CI [0.68, 1.16]) in the pitch-matching and rhythmic entrainment models, respectively.
501 Notably, the base model predicting task performance as a function of pitch-matching initially
502 failed to converge and required the bobyqa optimizer to reconcile this issue. Importantly, the
503 effect sizes for all inconsistent interpretations for time were trivial or small, ranging from a
504 Cohen’s d of 0.15 (Fiction Emotion-Matching performance vs Year), to d = 0.34 (IECA score vs.
505 Year). On the other hand, the effect sizes for time in the Eyes Test models were much larger at a
506 Cohen’s *d* of 0.81 to 0.92 and exhibited a shared finding across models given the effect’s
507 robustness.

# 508 Discussion

## 509 Pitch-matching and rhythmic entrainment development

510 The present longitudinal study aimed to explore the development of pitch-matching,
511 rhythmic entrainment, and socioemotional skills in a population of 6-year-old children enrolled
512 in either a music, sports, or no after-school program. We also aimed to identify associations
513 between our musical and socioemotional measures. view [111-114] and longitudinal studies [115], and even in
514 populations lacking formal music instruction [116]. Yet in most of these studies, participants
515 were asked to complete shorter tasks, often involving 2-3 pitches instead of longer pitch
516 melodies and scales, as in the present study. Child musicians in our study were primarily violin
517 and viola players, which are melodic instruments. Violinists and violists are used to playing short
518 melodies and practicing scales. We suggest that the improvements of child musicians on pitch-
519 matching over time is an indication of near transfer.
520 Analysis also revealed cognitive ability to have a significant yet small relationship with
521 pitch-matching. As evidenced by studies on the far-transfer effects of formal music instruction
522 on visuospatial (e.g., [117-119]) and verbal memory (e.g., [120-125]), musical ability appears to
523 robustly associate with general cognition. For instance, Berkowska and Dalla Bella [120] found
524 that reducing the linguistic complexity of a target stimulus decreased cognitive load,
525 subsequently improving pitch-matching performance. In line with their findings, individuals with
526 higher FSIQ-4 scores in our study were likely better equipped to store pitch information and later
527 reproduce them.
528 In contrast to pitch-matching, where time-related improvements were exclusive among
529 musicians, participants from all conditions significantly improved in their ability to rhythmically
530 entrain each year. Nevertheless, no musician’s advantage was found; the rate of improvement did
531 not change as a function of group. We speculate that this finding could be related to the nature of
532 music learning in an orchestral setting, particularly among beginning string players. It would be
533 interesting for future studies to study the development of rhythmic entrainment skills within
534 orchestral musicians, as there could be differences between instruments that typically play
535 melodic materials (e.g., violins, flutes) and those that help sustain the underlying beat of the
536 ensemble (e.g., percussion). Previous studies on the rhythmic entrainment skills of expert adult
537 instrumentalists have produced contrasting findings (e.g., [126, 127]), and research on
538 developing musicians may offer additional insights.
539 A common limitation in longitudinal studies is also the fragmented nature of data
540 collection. As studies continue, existing measurements are updated to be more age appropriate,
541 participants might be unavailable to test one year due to personal circumstances, and others may
542 lose interest and dropout from the study entirely. The present study is no stranger to these issues,
543 resulting in incomplete baseline pitch-matching and year 1 rhythmic entrainment data. It is
544 possible that the specific phrases of pitch-matching unmeasured at baseline (phrases 2 and 4) are
545 fundamentally more challenging for 6-year-old children to remember or produce, and that natural
546 improvements between the ages of 6 and 7 are substantial enough to alter the overall trajectory of
547 the model. This assumption is not unreasonable as phrases 2 and 4 were designed to incorporate
548 musical elements the AIRS Test Battery of Singing Skills (phrases 1 and 3) lacked, such as the
549 minor mode, articulation, and expressivity. Likewise, it could be argued that rhythmic
550 entrainment to a 180-bpm stimulus is a challenging task, particularly for child nonmusicians, as
551 it strays far away from a child’s spontaneous motor tempo [3, 14]. However, due to the absence
552 of year 1 data, we are unable to determine the training-related differences associated with
553 rhythmic entrainment at this tempo.
554 Of note, our analysis suggests that there may have been some baseline differences in the
555 measures of pitch-matching and rhythmic entrainment. Specifically, at baseline, participants in
556 the music program outperformed those in the sports group on pitch-matching while a group
557 difference between the music and control groups approached significance in the rhythmic
558 entrainment task. Combined with the fact that children self-enrolled into their programs, there is
559 a chance that these baseline differences are influenced by selection bias. Parents of children who
560 enrolled them in the music program may more likely have home environments that valued
561 musical exposure and more frequently participated in musical activities (e.g., singing/dancing at
562 home, attending musical events with family, etc.).
563 We note that Ilari, Fesjian, and Habibi [97] from our group analyzed the baseline
564 rhythmic entrainment data to the 120-bpm stimuli for the same dataset and found no significant
565 group differences in the alone condition; however, they did find a significant difference between
566 the music and control groups in the social condition. Similarly, Wood, Perez, et al. [128]
567 performed a PCA on the same pitch-matching and rhythmic entrainment data and report no
568 baseline differences in the resulting component they refer to as “music training”. We believe
569 these differences stem from the difference in the outcome variables and analysis models. The
570 dataset analyzed by Ilari et al. [97] is most conservative in their methods where they used
571 untransformed raw data and omitted all participants with missing data. In the report by Wood,
572 Perez et al. [128] they extracted a single construct of music training skills combining all
573 available data on rhythmic entrainment and pitch-matching. The present study acts as an
574 intermediary by analyzing a transformed variable representing overall performance for rhythmic
575 entrainment and pitch-matching separately. Similar to the findings by Ilari et al., which used raw
576 untransformed data for their analysis of baseline performance, we show no differences in the
577 alone condition for the 120-bpm stimuli, while a group difference between the music and control
578 group approached significance in the social condition. For the 180-bpm stimuli, musicians
579 significantly outperformed the control group in both the alone and social conditions. Our results
580 confirm that these group differences continue to hold over-time. As for pitch-matching using the
581 raw untransformed data, there were no group differences for phrase 1 of the pitch-matching task,
582 but that musicians did outperform the sports group for phrase 3. Despite this baseline difference,
583 the music group consistently improved overtime in their pitch-matching ability whereas the other
584 groups did not.

## 585 Socioemotional development

586 Children’s sharing behavior was found to improve over time. As explained by Malti et al.
587 [79], sharing is a costly behavior that rarely benefits the sharer. In one-off incidents like a
588 laboratory experiment, the incentive to share is often reduced as the potential rewards associated
589 with continued interaction are unavailable (e.g., building rapport with the recipient,
590 strengthening a relationship, etc.). Children in our study replicated the results from Malti et al. by
591 exhibiting an increase in sharing from ages eight to nine, followed by a decrease in sharing from
592 ages nine to 10. As observed by Benenson, Pascoe, and Radmore [100] it is not uncommon for
593 this transitional period between early and middle childhood to coincide with an increase in
594 sharing behavior. However, with continued growth in perspective-taking and additional
595 opportunities for peer interaction, children might instead become more mindful and selective
596 with whom they share with [129]. Interestingly, individuals in the sports programs shared at a
597 higher rate than the music group, which could be the result of more frequent exposure to
598 strangers during sports games and meets. In stark contrast, child musicians were rarely afforded
599 interaction with people outside of the music program (with the exception of authority figures
600 such as teachers and conductors). This may have prevented them from normalizing interactions
601 with new people.
602 A drawback to our design is that the prosocial task did not immediately follow rhythmic
603 entrainment. At each time point, the sharing tasks were the last to be administered; the rhythmic
604 entrainment and sharing tasks could be administered hours apart. Although some research has
605 identified a trait effect of rhythmic entrainment on socioemotional skills [130], many of the
606 previous studies on this topic have identified a more transient state effect of rhythmic
607 entrainment on socioemotional outcomes (e.g., [23], [56], [131]). It could be that rhythmic
608 entrainment induces a state effect, whereby musicians are more prosocial during and
609 immediately following rhythmic engagement. However, once they return to their everyday life,
610 these prosocial gains revert to normal levels. Moreover, the rhythmic entrainment and sharing
611 tasks were conducted between different pairs of individuals; the rhythmic entrainment task was
612 between the participant and the experiment while the sharing task was between the participant
613 and the representation (i.e., a paper bag) of an absent stranger or friend. The dynamics of this
614 interaction may have changed if the receiver was instead the experimenter, or if the receiver was
615 physically present.
616 Concerning the empathy domain, the growth curves for each measure followed separate
617 and unique patterns. Bryant’s IECA did not improve over time but did as a function of working
618 memory. Conversely, the Eyes Test improved drastically over time without an effect of cognitive
619 ability. Finally, the Fiction Emotion-Matching task was not moderated by any measure of
620 cognitive ability but did include a greater yearly improvement for musicians relative to athletes.
621 When interpreting these results, it is important to consider the intended purpose of each measure;
622 Bryant’s IECA broadly measures trait empathy (however, see [132]), the Eyes Test measures
623 theory of mind (however, see [133, 134]), and the Fiction Emotion-Matching task is a holistic
624 measure assessing multiple aspects of empathy (i.e., situational, cognitive, and theory of mind).
625 With regards to the IECA, Rabinowitch, Cross, and Burnard [84] found performance on
626 this task to improve due to formal music instruction. However, it is important to note that their
627 music intervention explicitly specialized in training empathic behaviors. Outside of this study,
628 there is limited evidence to support the notion that musicians are characteristically more
629 empathic than the general population, and this is reiterated in our results. Our findings are more
630 similar to those of Cespedes-Guevara and Dibben [94], who did not find improvements on
631 measures of trait or situational empathy. Both our study and Cespedes-Guevara and Dibben
632 focused on large ensemble programs, and more specifically, music learning in orchestras.
633 Although orchestral training programs have undergone some changes, they still tend to be highly
634 structured and hierarchical. It is possible that music programs predicated on collaboration such as
635 popular music, jazz and mariachi foster heightened empathy in developing musicians. This
636 would be consistent with studies that found differences in empathy scores favoring popular
637 musicians over classically-trained musicians [135].
638 As for the Eyes Test, this measure fundamentally requires participants to look into
639 another’s eyes and evaluate their emotional state. Very rarely do musicians probe for emotional
640 information from someone’s eyes alone; rather, eye-contact in a musical setting seems to be used
641 primarily for coordinating technical aspects of the performance (e.g., entries and synchronizing
642 rhythmic sections). In fact, studies tend to find null or mixed results when associating formal
643 music training with the recognition of emotion from visual [136] and audiovisual [48, 137, 138]
644 stimuli. In a longitudinal study conducted by Good et al. [48], small-n groups of children with
645 cochlear implants were enrolled into either a music or visual-arts program. After 6-months of
646 training, pre/post measures of auditory and audiovisual emotion-recognition were compared
647 yielding no significant differences between groups. Importantly, much of this research comes
648 from cross-sectional investigations of cochlear implant users, so additional longitudinal research
649 with larger populations of individuals with normal hearing are required before relationships can
650 be established.
651 Finally, success in the Fiction Emotion-Matching requires participants to identify the
652 emotion of a fictional character then emulate that same emotion. This task is unique among our
653 empathy measures as it incorporates an auditory dimension, of which musicians are known to
654 process differently than nonmusicians. In the context of our Fiction Emotion-Matching task,
655 musicians may be uniquely advantaged in utilizing their enhanced auditory processing to identify
656 emotions conveyed by a movie soundtrack or a speaker’s words. Although our study did not
657 specifically assess auditory emotion recognition, we speculate that similar mechanisms may be at
658 play — a musician’s heightened sensitivity to emotional cues in auditory stimuli may lead to an
659 improvement in identifying and ultimately embody the emotions conveyed in a movie clip.
660 Additional covariates such as a general cognitive ability and working memory were also
661 expected to moderate our outcome variables, as previous studies have demonstrated a strong,
662 positive relationship between formal music instruction and cognitive ability [139-140].
663 Schellenberg and Mankarious [141], for example, found formal music instruction to improve
664 emotional comprehension; however, this effect disappeared when FSIQ scores were held
665 constant. In the present study, effects of cognitive ability on model estimates were generally
666 observed, but with a much weaker effect than anticipated. Similarly, although trending and even
667 significant associations were found between socioemotional skills and music production abilities,
668 each of these effect sizes were marginal to small in magnitude. Overall, time appears to be the
669 most frequent and robust predictor for any improvements on socioemotional behaviors, which
670 we come to expect over the natural course of human development and maturation.
671 The results of this study provide limited support for previous literature associating
672 socioemotional abilities, formal music instruction, and their interaction (e.g., [84], [93], [97]).
673 Ilari et al. [97], which used the same data as this study, albeit at one individual time point, found
674 a positive correlation between rhythmic entrainment ability and prosocial behavior in the music
675 group. Although this correlation was strong, the relationship was only present between scores of
676 rhythmic entrainment with a metronome (in the alone condition) and the number of stickers
677 given to a friend, not a stranger. This study offers a more holistic assessment of the data by
678 creating composite measures of rhythmic entrainment in all conditions, as well as scoring
679 prosociality through the total proportion of stickers given away (e.g., [79]) instead of splitting it
680 by the receiver. Although splitting the data by the receiver would allow for a more nuanced
681 perspective into the sharing behavior of children, combining the conditions as we did here allow
682 us to assess sharing behavior across a longer timeframe.
683 Our research also contributes to the growing body of literature identifying a relationship
684 between formal music instruction and socioemotional gains in the auditory rather than visual
685 modality. Much of the literature focuses on the identification of emotional states elicited by the
686 face, except whole-body gestures remain a key aspect of musical engagement (e.g., bobbing and
687 swaying, [142]). It would be interesting for future research to evaluate a musician’s ability for
688 emotion recognition from body language. This will help clarify whether their advantage is
689 limited to the auditory domain. Moreover, while it is not uncommon to assess socioemotional
690 skills in relation to formal music instruction, previous research tends to measure a comparatively
691 short timeframe (e.g., 6-months, [22]; 9-months, [84]; 1-year, [94]). Yet, musical development is
692 a lifelong process. Assessing behavioral outcomes from formal music instruction within a single
693 year could be premature. Likewise, it is important to consider that any socioemotional outcomes
694 derived from participation in a formal music program are dependent on the quality and objectives
695 of the program. Our study focused on a music program based on the orchestral model, and placed
696 emphasis on the development of musical skills through an intensive training process. While the
697 program did foster some social and collaborative skills, the primary goal of the program was not
698 the development of socioemotional skills as in Rabinowitch et al. [84], nor to compare the
699 experiences of musicians who practiced different genres including popular music [135, 143]. By
700 measuring musical and socioemotional skills over the course of multiple years, we are uniquely
701 positioned to capture the long-term growth resulting from formal music instruction and offer
702 robust data on the complex construct that is musical development.

# 703 Acknowledgements

704 We are grateful to Priscilla Perez and Alison Wood for their contributions to this project.

# 705 References

706 1. Amir O, Amir N, Kishon-Rabin L. The effect of superior auditory skills on vocal accuracy. J

713	Acoust Soc Am. 2003 Feb;113(2):1102-8. doi: [10.1121/1.1536632](https://doi.org/10.1121/1.1536632)

714	2. Bilhartz TD, Bruhn RA, Olson JE. The effect of early music training on child cognitive

715	development. Journal of Applied Developmental Psychology. 1999 Dec 1;20(4):615-36. doi:

716	[10.1016/s0193-3973(99)00033-7](https://doi.org/10.1016/s0193-3973(99)00033-7)

717	3. Drake C, Jones MR, Baruch C. The development of rhythmic attending in auditory

718	sequences: theory and research. Cognition. 2000;77(3):251-88. doi: [10.1016/s0010-](https://doi.org/10.1016/s0010-0277(00)00106-2)

719	[0277(00)00106-2](https://doi.org/10.1016/s0010-0277(00)00106-2)

720	4. Pfordresher PQ, Demorest SM. The prevalence and correlates of accurate singing. Journal of

721	Research in Music Education. 2021 Apr;69(1):5-23. doi: [10.1177/0022429420951630](https://doi.org/10.1177/0022429420951630)

722	5. Repp BH, Su YH. Sensorimotor synchronization: a review of recent research (2006-2012).

723	Psychon Bull Rev. 2013 Jun;20(3):403-52. doi: [10.3758/s13423-012-0371-2](https://doi.org/10.3758/s13423-012-0371-2)

724	6. Berkowska M, Dalla Bella S. Acquired and congenital disorders of sung performance: A

725	review. Adv Cogn Psychol. 2009 Nov 12;5:69-83. doi: [10.2478/v10053-008-0068-2](https://doi.org/10.2478%2Fv10053-008-0068-2)

726	7. Dalla Bella S, Giguère JF, Peretz I. Singing proficiency in the general population. J Acoust

727	Soc Am. 2007 Feb;121(2):1182-9. doi: [10.1121/1.2427111](https://doi.org/10.1121/1.2427111)

728	8. Trollinger VL. Relationships between pitch-matching accuracy, speech fundamental

729	frequency, speech range, age, and gender in American English-speaking preschool children.

730	Journal of Research in Music Education. 2003 Apr;51(1):78-94. doi: [10.2307/3345650](https://doi.org/10.2307/3345650)

731	9. McPherson G, editors. The child as musician: A handbook of musical development. Oxford

732	University Press; 2015. p. 311-330. doi: 10.1093/acprof:oso/9780198530329.003.0016

| 733 | 10. Bennett M, Schatz MF, Rockwood H, Wiesenfeld K. Huygens's clocks. Proceedings of the |
| --- | --- |
| 734 | Royal Society of London. Series A: Mathematical, Physical and Engineering Sciences. 2002 |
| 735 | Mar;458(2019):563-79. doi: [10.1098/rspa.2001.0888](http://doi.org/10.1098/rspa.2001.0888) |
| 736 | 11. Ermentrout B. An adaptive model for synchrony in the firefly Pteroptyx malaccae. Journal of |
| 737 | Mathematical Biology. 1991 Jun;29(6):571-85. doi: [10.1007/BF00164052](http://doi.org/10.1007/BF00164052) |
| 738 | 12. Phillips-Silver J, Trainor LJ. Feeling the beat: movement influences infant rhythm |
| 739 | perception. Science. 2005 Jun 3;308(5727):1430. doi: [10.1126/science.1110922](https://doi.org/10.1126/science.1110922) |
| 740 | 13. Ilari B. Rhythmic engagement with music in early childhood: A replication and extension. |
| 741 | Journal of Research in Music Education. 2015 Jan;62(4):332-343. doi: |
| 742 | [10.1177/0022429414555984](https://doi.org/10.1177/0022429414555984) |
| 743 | 14. McAuley JD, Jones MR, Holub S, Johnston HM, Miller NS. The time of our lives: life span |
| 744 | development of timing and event tracking. J Exp Psychol Gen. 2006 Aug;135(3):348-67. doi: |
| 745 | [10.1037/0096-3445.135.3.348](https://doi.org/10.1037/0096-3445.135.3.348) |
| 746 | 15. Zentner M, Eerola T. Rhythmic engagement with music in infancy. Proc Natl Acad Sci U S |
| 747 | A. 2010 Mar 30;107(13):5768-73. doi: [10.1073/pnas.1000121107](https://doi.org/10.1073/pnas.1000121107) |
| 748 | 16. Phillips-Silver J, Aktipis CA, Bryant GA. The ecology of entrainment: Foundations of |
| 749 | coordinated rhythmic movement. Music Percept. 2010 Sep;28(1):3-14. doi: |
| 750 | [10.1525/mp.2010.28.1.3](https://doi.org/10.1525/mp.2010.28.1.3) |
| 751 | 17. Levitin DJ, Grahn JA, London J. The Psychology of Music: Rhythm and Movement. Annu |
| 752 | Rev Psychol. 2018 Jan 4;69:51-75. doi: [10.1146/annurev-psych-122216-011740](https://doi.org/10.1146/annurev-psych-122216-011740) |
| 753 | 18. McNeill WH. Keeping together in time: Dance and drill in human history. Harvard |
| 754 | University Press; 1997 Oct 30. doi: [10.4159/9780674040878](http://doi.org/10.4159/9780674040878) |

755	19. Greenfield MD. Mechanisms and evolution of communal sexual displays in arthropods and

756	anurans. Advances in the Study of Behavior. 2005 Jan 1;35(05):1-62. doi: [10.1016/s0065-](https://doi.org/10.1016/s0065-3454(05)35001-7)

757	[3454(05)35001-7](https://doi.org/10.1016/s0065-3454(05)35001-7)

758	20. Ravignani A, Bowling DL, Fitch WT. Chorusing, synchrony, and the evolutionary functions

759	of rhythm. Front Psychol. 2014 Oct 10;5:1118. doi: [10.3389/fpsyg.2014.01118](https://doi.org/10.3389/fpsyg.2014.01118)

760	21. Greenspon EB, Montanaro V. Singing ability is related to vocal emotion recognition:

761	Evidence for shared sensorimotor processing across speech and music. Atten Percept

762	Psychophys. 2023 Jan;85(1):234-243. doi: [10.3758/s13414-022-02613-0](https://doi.org/10.3758/s13414-022-02613-0)

763	22. Good A, Russo FA. Singing promotes cooperation in a diverse group of children. Social

764	Psychology. 2016 Oct;47(6):340-4. doi: [10.1027/1864-9335/a000282](https://doi.org/10.1027/1864-9335/a000282)

765	23. Kirschner S, Tomasello M. Joint music making promotes prosocial behavior in 4-year-old

766	children. Evolution and Human Behavior. 2010 Sep 1;31(5):354-64. doi:

767	[10.1016/j.evolhumbehav.2010.04.004](https://doi.org/10.1016/j.evolhumbehav.2010.04.004)

768	24. Wiltermuth SS, Heath C. Synchrony and cooperation. Psychol Sci. 2009 Jan;20(1):1-5. doi:

769	[10.1111/j.1467-9280.2008.02253.x](https://doi.org/10.1111/j.1467-9280.2008.02253.x)

770	25. Eisenberg N, Spinrad TL, Morris AS. Prosocial Development 13. The Oxford Handbook of

771	Developmental Psychology, Vol. 2: Self and Other. 2013 Jan 15;2:300.

772	26. Hove MJ, Risen JL. It's all in the timing: Interpersonal synchrony increases affiliation. Social

773	Cognition. 2009 Dec;27(6):949-60. doi: [10.1521/soco.2009.27.6.949](https://doi.org/10.1521/soco.2009.27.6.949)

774 27. Tarr B, Launay J, Cohen E, Dunbar R. Synchrony and exertion during dance independently
775 raise pain threshold and encourage social bonding. Biol Lett. 2015 Oct;11(10):20150767.
776 doi: [10.1098/rsbl.2015.0767](https://doi.org/10.1098/rsbl.2015.0767)
777 28. Tarr B, Launay J, Dunbar RI. Silent disco: dancing in synchrony leads to elevated pain
778 thresholds and social closeness. Evol Hum Behav. 2016 Sep;37(5):343-349. doi:
779 [10.1016/j.evolhumbehav.2016.02.004](https://doi.org/10.1016/j.evolhumbehav.2016.02.004)
780 29. Giles H, Coupland N, Coupland J. Accommodation theory: Communication, context, and
781 consequence. In: Giles H, Coupland J, Coupland N, editors. Contexts of Accommodation:
782 Developments in Applied Sociolinguistics. Cambridge: Cambridge University Press; 1991. p.
783 1–68. doi: [10.1017/cbo9780511663673.001](https://doi.org/10.1017/cbo9780511663673.001)
784 30. Pickering MJ, Ferreira VS. Structural priming: a critical review. Psychol Bull. 2008

785	May;134(3):427-59. doi: [10.1037/0033-2909.134.3.427](https://psycnet.apa.org/doi/10.1037/0033-2909.134.3.427)

786	31. von Zimmermann J, Richardson DC. Verbal Synchrony and Action Dynamics in Large

787	Groups. Front Psychol. 2016 Dec 26;7:2034. doi: [10.3389/fpsyg.2016.02034](https://doi.org/10.3389/fpsyg.2016.02034)

788 32. De Looze C, Oertel C, Rauzy S, Campbell N. Measuring dynamics of mimicry by means of
789 prosodic cues in conversational speech. Proceedings of the 17th International Congress of
790 Phonetic Sciences; 2011 August 17-21; Hong Kong, China.
791 33. De Looze C, Scherer S, Vaughan B, Campbell N. Investigating automatic measurements of
792 prosodic accommodation and its dynamics in social interaction. Speech Communication.
793 2014 Mar 1;58:11-34. doi: [10.1016/j.specom.2013.10.002](https://doi.org/10.1016/j.specom.2013.10.002)
794 34. Imel ZE, Barco JS, Brown HJ, Baucom BR, Baer JS, Kircher JC, Atkins DC. The association
795 of therapist empathy and synchrony in vocally encoded arousal. J Couns Psychol. 2014
796 Jan;61(1):146-53. doi: [10.1037/a0034943](https://doi.org/10.1037/a0034943)
797 35. Michalsky J. Pitch synchrony as an effect of perceived attractiveness and likability.
798 Proceedings of the 43rd Annual Conference on Acoustics; 2017 March 6-9; Kiel, Germany
799 36. Michalsky, J., Schoormann, H. (2017) Pitch Convergence as an Effect of Perceived
800 Attractiveness and Likability. Proceedings of the 18th Annual Conference of the
801 International Speech Communication Association; 2017 Aug 20-24; Stockholm, Sweden. p.
802 2253-2256, doi: 10.21437/Interspeech.2017-1520
803 37. Lubold N, Pon-Barry H. Acoustic-prosodic entrainment and rapport in collaborative learning
804 dialogues. Proceedings of the 2014 ACM Workshop on Multimodal Learning Analytics
805 Workshop and Grand Challenge; 2014 Nov 12; Istanbul, Turkey. p. 5-12. doi:
806 [10.1145/2666633.2666635](https://doi.org/10.1145/2666633.2666635)
807 38. Lee CC, Black M, Katsamanis A, Lammert AC, Baucom BR, Christensen A, Georgiou PG,
808 Narayanan SS. Quantification of prosodic entrainment in affective spontaneous spoken
809 interactions of married couples. Proceedings of the 11th Annual Conference of the
810 International Speech Communication Association; 2010 September 26-30; Makuhari, Chiba,
811 Japan. p. 793-796. doi: [10.21437/interspeech.2010-287](https://doi.org/10.21437/interspeech.2010-287)
812 39. Neves L, Correia AI, Castro SL, Martins D, Lima CF. Does music training enhance auditory
813 and linguistic processing? A systematic review and meta-analysis of behavioral and brain
814 evidence. Neurosci Biobehav Rev. 2022 Sep;140:104777. doi:
815 [10.1016/j.neubiorev.2022.104777](https://doi.org/10.1016/j.neubiorev.2022.104777)
816 40. Besson M, Schön D, Moreno S, Santos A, Magne C. Influence of musical expertise and
817 musical training on pitch processing in music and language. Restorative neurology and
818 neuroscience. 2007 Jan 1;25(3-4):399-410. PMID: [17943015](https://www.ncbi.nlm.nih.gov/pubmed/17943015)
819 41. Habibi A, Cahn BR, Damasio A, Damasio H. Neural correlates of accelerated auditory
820 processing in children engaged in music training. Developmental Cognitive Neuroscience.
821 2016 Oct;21:1-4. doi: [10.1016/j.dcn.2016.04.003](https://doi.org/10.1016/j.dcn.2016.04.003)
822 42. Schellenberg EG, Moreno S. Music lessons, pitch processing, and g. Psychology of Music.

823	2010 Apr;38(2):209-21. doi: [10.1177/0305735609339473](https://doi.org/10.1177/0305735609339473)

824 43. Chen JL, Penhune VB, Zatorre RJ. Moving on time: brain network for auditory-motor
825 synchronization is modulated by rhythm complexity and musical training. Journal of
826 Cognitive Neuroscience. 2008 Feb 1;20(2):226-39. doi: [10.1162/jocn.2008.20018](https://doi.org/10.1162/jocn.2008.20018)
827 44. Fiveash A, Bedoin N, Gordon RL, Tillmann B. Processing rhythm in speech and music:
828 Shared mechanisms and implications for developmental speech and language disorders.
829 Neuropsychology. 2021 Nov;35(8):771. doi: [10.1037/neu0000766](https://psycnet.apa.org/doi/10.1037/neu0000766)
830 45. Repp BH. Sensorimotor synchronization and perception of timing: effects of music training
831 and task experience. Human Movement Science. 2010 Apr 1;29(2):200-13. doi:
832 [10.1016/j.humov.2009.08.002](https://doi.org/10.1016/j.humov.2009.08.002)
833 46. Putkinen V, Tervaniemi M, Huotilainen M. Musical playschool activities are linked to faster
834 auditory development during preschool-age: a longitudinal ERP study. Scientific Reports.
835 2019 Aug 5;9(1):11310. doi: [10.1038/s41598-019-47467-z](https://doi.org/10.1038/s41598-019-47467-z)
836 47. Fuller CD, Galvin JJ 3rd, Maat B, Free RH, Başkent D. The musician effect: does it persist
837 under degraded pitch conditions of cochlear implant simulations? Front Neurosci. 2014 Jun
838 30;8:179. doi: [10.3389/fnins.2014.00179](https://doi.org/10.3389%2Ffnins.2014.00179)
839 48. Good A, Gordon KA, Papsin BC, Nespoli G, Hopyan T, Peretz I, Russo FA. Benefits of
840 Music Training for Perception of Emotional Speech Prosody in Deaf Children With Cochlear
841 Implants. Ear Hear. 2017 Jul/Aug;38(4):455-464. doi: [10.1097/AUD.0000000000000402](https://doi.org/10.1097%2FAUD.0000000000000402)
842 49. Lima CF, Castro SL. Speaking to the trained ear: musical expertise enhances the recognition
843 of emotions in speech prosody. Emotion. 2011 Oct;11(5):1021-31. doi: [10.1037/a0024521](https://psycnet.apa.org/doi/10.1037/a0024521)
844 50. Thompson WF, Schellenberg EG, Husain G. Decoding speech prosody: do music lessons

845	help? Emotion. 2004 Mar;4(1):46-64. doi: [10.1037/1528-3542.4.1.46](https://psycnet.apa.org/doi/10.1037/1528-3542.4.1.46)

846 51. Trimmer CG, Cuddy LL. Emotional intelligence, not music training, predicts recognition of
847 emotional speech prosody. Emotion. 2008 Dec;8(6):838-49. doi: [10.1037/a0014080](https://psycnet.apa.org/doi/10.1037/a0014080)
848 52. Dellacherie D, Roy M, Hugueville L, Peretz I, Samson S. The effect of musical experience
849 on emotional self-reports and psychophysiological responses to dissonance.
850 Psychophysiology. 2011 Mar;48(3):337-49. doi: [10.1111/j.1469-8986.2010.01075.x](https://doi.org/10.1111/j.1469-8986.2010.01075.x)
851 53. Mikutta CA, Maissen G, Altorfer A, Strik W, Koenig T. Professional musicians listen
852 differently to music. Neuroscience. 2014 May 30;268:102-11. doi:
853 [10.1016/j.neuroscience.2014.03.007](https://doi.org/10.1016/j.neuroscience.2014.03.007)
854 54. Park M, Gutyrchik E, Bao Y, Zaytseva Y, Carl P, Welker L, Pöppel E, Reiser M, Blautzik J,
855 Meindl T. Differences between musicians and non-musicians in neuro-affective processing
856 of sadness and fear expressed in music. Neurosci Lett. 2014 Apr 30;566:120-4. doi:
857 [10.1016/j.neulet.2014.02.041](https://doi.org/10.1016/j.neulet.2014.02.041)
858 55. Mogan R, Fischer R, Bulbulia JA. To be in synchrony or not? A meta-analysis of
859 synchrony's effects on behavior, perception, cognition and affect. Journal of Experimental
860 Social Psychology. 2017 Sep 1;72:13-20. doi: [10.1016/j.jesp.2017.03.009](https://doi.org/10.1016/j.jesp.2017.03.009)
861 56. Tunçgenç B, Cohen E. Movement Synchrony Forges Social Bonds across Group Divides.

862	Front Psychol. 2016 May 27;7:782. doi: [10.3389/fpsyg.2016.00782](https://doi.org/10.3389/fpsyg.2016.00782)

863 57. Rabinowitch TC, Knafo-Noam A. Synchronous rhythmic interaction enhances children's
864 perceived similarity and closeness towards each other. PLoS One. 2015 Apr
865 8;10(4):e0120878. doi: [10.1371/journal.pone.0120878](https://doi.org/10.1371/journal.pone.0120878)
866 58. Kirschner S, Ilari B. Joint drumming in Brazilian and German preschool children: Cultural
867 differences in rhythmic entrainment, but no prosocial effects. Journal of Cross-Cultural
868 Psychology. 2014 Jan;45(1):137-66. doi: [10.1177/0022022113493139](https://doi.org/10.1177/0022022113493139)
869 59. Parbery-Clark A, Skoe E, Kraus N. Musical experience limits the degradative effects of
870 background noise on the neural processing of sound. J Neurosci. 2009 Nov 11;29(45):14100-
871 7. doi: [10.1523/jneurosci.3256-09.2009](https://doi.org/10.1523/jneurosci.3256-09.2009)
872 60. Hennessy SL, Sachs ME, Ilari B, Habibi A. Effects of Music Training on Inhibitory Control
873 and Associated Neural Networks in School-Aged Children: A Longitudinal Study. Front
874 Neurosci. 2019 Oct 16;13:1080. doi: [10.3389/fnins.2019.01080](https://doi.org/10.3389/fnins.2019.01080)
875 61. Southgate DE, Roscigno VJ. The impact of music on childhood and adolescent achievement.

876	Social Science Quarterly. 2009 Mar;90(1):4-21. doi: [10.1111/j.1540-6237.2009.00598.x](https://doi.org/10.1111/j.1540-6237.2009.00598.x)

877	62. Hetland L. Learning to make music enhances spatial reasoning. Journal of aesthetic

878	education. 2000 Oct 1;34(3/4):179-238. doi: [10.2307/3333643](https://doi.org/10.2307/3333643)

879	63. Köster M, Cavalcante L, Vera Cruz de Carvalho R, Dôgo Resende B, Kärtner J. Cultural

880	Influences on Toddlers' Prosocial Behavior: How Maternal Task Assignment Relates to

881	Helping Others. Child Dev. 2016 Nov;87(6):1727-1738. doi: [10.1111/cdev.12636](https://doi.org/10.1111/cdev.12636)

882	64. Wellman HM, Cross D, Watson J. Meta-analysis of theory-of-mind development: the truth

883	about false belief. Child Dev. 2001 May-Jun;72(3):655-84. doi: [10.1111/1467-8624.00304](https://doi.org/10.1111/1467-8624.00304)

884 65. Osterhaus C, Koerber S. The Development of Advanced Theory of Mind in Middle
885 Childhood: A Longitudinal Study From Age 5 to 10 Years. Child Dev. 2021 Sep;92(5):1872-
886 1888. doi: [10.1111/cdev.13627](https://doi.org/10.1111/cdev.13627)
887 66. Burnett S, Bird G, Moll J, Frith C, Blakemore SJ. Development during adolescence of the
888 neural processing of social emotion. J Cogn Neurosci. 2009 Sep;21(9):1736-50. doi:
889 [10.1162/jocn.2009.21121](https://doi.org/10.1162/jocn.2009.21121)
890 67. Pfeifer JH, Lieberman MD, Dapretto M. "I know you are but what am I?!": neural bases of
891 self- and social knowledge retrieval in children and adults. J Cogn Neurosci. 2007
892 Aug;19(8):1323-37. doi: [10.1162/jocn.2007.19.8.1323](https://doi.org/10.1162/jocn.2007.19.8.1323)
893 68. Pfeifer JH, Masten CL, Borofsky LA, Dapretto M, Fuligni AJ, Lieberman MD. Neural
894 correlates of direct and reflected self-appraisals in adolescents and adults: when social
895 perspective-taking informs self-perception. Child Dev. 2009 Jul-Aug;80(4):1016-38. doi:
896 [10.1111/j.1467-8624.2009.01314.x](https://doi.org/10.1111/j.1467-8624.2009.01314.x)
897 69. Blakemore SJ, den Ouden H, Choudhury S, Frith C. Adolescent development of the neural
898 circuitry for thinking about intentions. Soc Cogn Affect Neurosci. 2007 Jun;2(2):130-9. doi:
899 [10.1093/scan/nsm009](https://doi.org/10.1093/scan/nsm009)
900 70. Fletcher PC, Happé F, Frith U, Baker SC, Dolan RJ, Frackowiak RS, Frith CD. Other minds
901 in the brain: a functional imaging study of "theory of mind" in story comprehension.
902 Cognition. 1995 Nov;57(2):109-28. doi: [10.1016/0010-0277(95)00692-R](https://doi.org/10.1016/0010-0277(95)00692-R)
903 71. Gallagher HL, Happé F, Brunswick N, Fletcher PC, Frith U, Frith CD. Reading the mind in
904 cartoons and stories: an fMRI study of 'theory of mind' in verbal and nonverbal tasks.
905 Neuropsychologia. 2000;38(1):11-21. doi: [10.1016/S0028-3932(99)00053-6](https://doi.org/10.1016/S0028-3932(99)00053-6)
906 72. Frith CD. The social brain? Philos Trans R Soc Lond B Biol Sci. 2007 Apr

907	29;362(1480):671-8. doi: [10.1098/rstb.2006.2003](https://doi.org/10.1098/rstb.2006.2003)

908	73. Saxe R. Uniquely human social cognition. Curr Opin Neurobiol. 2006 Apr;16(2):235-9. doi:

909	[10.1016/j.conb.2006.03.001](https://doi.org/10.1016/j.conb.2006.03.001)

910	74. Rowe AD, Bullock PR, Polkey CE, Morris RG. "Theory of mind" impairments and their

911	relationship to executive functioning following frontal lobe excisions. Brain. 2001

912	Mar;124(Pt 3):600-16. doi: [10.1093/brain/124.5.1062](https://doi.org/10.1093/brain/124.5.1062)

913	75. Stuss DT, Gallup GG Jr, Alexander MP. The frontal lobes are necessary for 'theory of mind'.

914	Brain. 2001 Feb;124(Pt 2):279-86. doi: [10.1093/brain/124.2.279](https://doi.org/10.1093/brain/124.2.279)

915 76. Samson D, Apperly IA, Chiavarino C, Humphreys GW. Left temporoparietal junction is
916 necessary for representing someone else's belief. Nat Neurosci. 2004 May;7(5):499-500. doi:
917 [10.1038/nn1223](https://doi.org/10.1038/nn1223)
918 77. Mills KL, Lalonde F, Clasen LS, Giedd JN, Blakemore SJ. Developmental changes in the
919 structure of the social brain in late childhood and adolescence. Soc Cogn Affect Neurosci.
920 2014 Jan;9(1):123-31. doi: [10.1093/scan/nss113](https://doi.org/10.1093/scan/nss113)
921 78. Nagaoka J, Farrington CA, Ehrlich SB, Heath RD. Developmental Progression Toward
922 Young Adulthood. In: Foundations for Young Adult Success: A Developmental Framework.
923 Concept Paper for Research and Practice. University of Chicago Consortium on Chicago
924 School Research; 2015 Jun. p. 57-76
925 79. Malti T, Ongley SF, Peplak J, Chaparro MP, Buchmann M, Zuffianò A, Cui L. Children's
926 Sympathy, Guilt, and Moral Reasoning in Helping, Cooperation, and Sharing: A 6-Year
927 Longitudinal Study. Child Dev. 2016 Nov;87(6):1783-1795. doi: [10.1111/cdev.12632](https://doi.org/10.1111/cdev.12632)
928 80. Clarke E, DeNora T, Vuoskoski J. Music, empathy and cultural understanding. Phys Life

929	Rev. 2015 Dec;15:61-88. doi: [10.1016/j.plrev.2015.09.001](https://doi.org/10.1016/j.plrev.2015.09.001)

930	81. Ilari B. Music in the early years: Pathways into the social world. Research Studies in Music

931	Education. 2016 Jun;38(1):23-39. doi: [10.1177/1321103x16642631](https://doi.org/10.1177/1321103x16642631)

| 932 | 82. Beck SL, Rieser J. Non-random acts of kindness: Joint music making increases preschoolers’ |
| --- | --- |
| 933 | helping and sharing with an adult. Psychology of Music. 2022 Jan;50(1):17-33. doi: |
| 934 | [10.1177/0305735620978688](https://doi.org/10.1177/0305735620978688) |
| 935 | 83. Rickard NS, Appelman P, James R, Murphy F, Gill A, Bambrick C. Orchestrating life skills: |
| 936 | The effect of increased school-based music classes on children’s social competence and self- |
| 937 | esteem. International Journal of Music Education. 2013 Aug;31(3):292-309. doi: |
| 938 | [10.1177/0255761411434824](https://doi.org/10.1177/0255761411434824) |
| 939 | 84. Rabinowitch TC, Cross I, Burnard P. Long-term musical group interaction has a positive |
| 940 | influence on empathy in children. Psychology of Music. 2013 Jul;41(4):484-98. doi: |
| 941 | [10.1177/0305735612440609](https://doi.org/10.1177/0305735612440609) |
| 942 | 85. Costa-Giomi E. Effects of three years of piano instruction on children’s academic |
| 943 | achievement, school performance and self-esteem. Psychology of Music. 2004 |
| 944 | Apr;32(2):139-52. doi: [10.1177/0305735604041491](https://doi.org/10.1177/0305735604041491) |
| 945 | 86. Rickard NS, Bambrick CJ, Gill A. Absence of widespread psychosocial and cognitive effects |
| 946 | of school-based music instruction in 10–13-year-old students. International Journal of Music |
| 947 | Education. 2012 Feb;30(1):57-78. doi: [10.1177/0255761411431399](https://doi.org/10.1177/0255761411431399) |
| 948 | 87. Portowitz A, Lichtenstein O, Egorova L, Brand E. Underlying mechanisms linking music |
| 949 | education and cognitive modifiability. Research Studies in Music Education. 2009 |
| 950 | Dec;31(2):107-28. doi: [10.1177/1321103x09344378](https://doi.org/10.1177/1321103x09344378) |
| 951 | 88. Schellenberg EG. Music lessons enhance IQ. Psychol Sci. 2004 Aug;15(8):511-4. doi: |
| 952 | [10.1111/j.0956-7976.2004.00711.x](https://doi.org/10.1111/j.0956-7976.2004.00711.x) |
| 953 | 89. Schellenberg EG. Long-term positive associations between music lessons and IQ. Journal of |
| 954 | Educational Psychology. 2006 May;98(2):457-468. doi: [10.1037/0022-0663.98.2.457](https://doi.org/10.1037/0022-0663.98.2.457) |

| 955 | 90. Anshel A, Kipper DA. The influence of group singing on trust and cooperation. Journal of |
| --- | --- |
| 956 | Music Therapy. 1988 Oct 1;25(3):145-55. doi: [10.1093/jmt/25.3.145](https://doi.org/10.1093/jmt/25.3.145) |
| 957 | 91. Gerry D, Unrau A, Trainor LJ. Active music classes in infancy enhance musical, |
| 958 | communicative and social development. Dev Sci. 2012 May;15(3):398-407. doi: |
| 959 | [10.1111/j.1467-7687.2012.01142.x](https://doi.org/10.1111/j.1467-7687.2012.01142.x) |
| 960 | 92. Boal-Palheiros G, Ilari B. Music, drama, and social development in Portuguese children. |
| 961 | Front Psychol. 2023 Jun 1;14:1093832. doi: [10.3389.fpsyg.2023.1093832](https://doi.org/10.3389.fpsyg.2023.1093832) |
| 962 | 93. Schellenberg EG, Corrigall KA, Dys SP, Malti T. Group Music Training and Children's |
| 963 | Prosocial Skills. PLoS One. 2015 Oct 27;10(10):e0141449. doi: |
| 964 | [10.1371/journal.pone.0141449](https://doi.org/10.1371/journal.pone.0141449) |
| 965 | 94. Cespedes-Guevara J, Dibben N. Promoting prosociality in Colombia: Is music more effective |
| 966 | than other cultural interventions? Music Sci. 2021 Sep;25(3):332-357. doi: |
| 967 | [10.1177/10298649211013505](https://doi.org/10.1177/10298649211013505) |
| 968 | 95. Pfordresher PQ, Brown S. Poor-pitch singing in the absence of" tone deafness". Music |
| 969 | Perception. 2007 Dec 1;25(2):95-115. doi: [10.1525/mp.2007.25.2.95](https://doi.org/10.1525/mp.2007.25.2.95) |
| 970 | 96. Cohen AJ, Armstrong VL, Lannan MS, Coady JD. A protocol for cross-cultural research on |
| 971 | the acquisition of singing. Ann N Y Acad Sci. 2009 Jul;1169:112-5. doi: [10.1111/j.1749-](https://doi.org/10.1111/j.1749-6632.2009.04771.x) |
| 972 | [6632.2009.04771.x](https://doi.org/10.1111/j.1749-6632.2009.04771.x) |
| 973 | 97. Ilari B, Fesjian C, Habibi A. Entrainment, theory of mind, and prosociality in child |
| 974 | musicians. Music & Science. 2018 Feb 15;1. doi: [10.1177/2059204317753153](https://doi.org/10.1177/2059204317753153) |
| 975 | 98. Kirschner S, Tomasello M. Joint drumming: social context facilitates synchronization in |
| 976 | preschool children. J Exp Child Psychol. 2009 Mar;102(3):299-314. doi: |
| 977 | [10.1016/j.jecp.2008.07.005](https://doi.org/10.1016/j.jecp.2008.07.005) |

| 978 | 99. MATLAB. Version 9.13.0 (R2022b), Natick, Massachusetts: The MathWorks Inc.; 2022. |
| --- | --- |
| 979 | 100. Benenson JF, Pascoe J, Radmore N. Children's altruistic behavior in the dictator game. |
| 980 | Evolution and Human Behavior. 2007 May 1;28(3):168-75. doi: |
| 981 | [10.1016/j.evolhumbehav.2006.10.003](https://doi.org/10.1016/j.evolhumbehav.2006.10.003) |
| 982 | 101. Bryant BK. An index of empathy for children and adolescents. Child Dev. 1982 Apr |
| 983 | 1;53(2):413-25. doi: [10.2307/1128984](https://doi.org/10.2307/1128984) |
| 984 | 102. Baron-Cohen S, Wheelwright S, Hill J, Raste Y, Plumb I. The "Reading the Mind in the |
| 985 | Eyes" Test revised version: a study with normal adults, and adults with Asperger syndrome |
| 986 | or high-functioning autism. J Child Psychol Psychiatry. 2001 Feb;42(2):241-51. PMID: |
| 987 | [11280420](https://pubmed.ncbi.nlm.nih.gov/11280420/). |
| 988 | 103. Goldstein TR, Winner E. Enhancing empathy and theory of mind. Journal of Cognition |
| 989 | and Development. 2012 Jan 1;13(1):19-37. doi: [10.1080/15248372.2011.573514](https://doi.org/10.1080/15248372.2011.573514) |
| 990 | 104. Wechsler, D. Wechsler Abbreviated Scale of Intelligence (WASI). San Antonio (TX): |
| 991 | Pearson; 1999. doi: [10.1037/t15170-000](https://psycnet.apa.org/doi/10.1037/t15170-000) |
| 992 | 105. R Core Team. R: A Language and Environment for Statistical Computing. Version 4.2.1 |
| 993 | [software]. Vienna, Austria; 2016 [cited 2023 Aug 23]. Available from: [https://www.R-](https://www.r-project.org/) |
| 994 | [project.org/](https://www.r-project.org/) |
| 995 | 106. Wickham H, Averick M, Bryan J, Chang W, McGowan LD, François R, Grolemund G, |
| 996 | Hayes A, Henry L, Hester J, Kuhn M. Welcome to the Tidyverse. Journal of Open Source |
| 997 | software. 2019 Nov 21;4(43):1686. doi: [10.21105/joss.01686](http://doi.org/10.21105/joss.01686) |
| 998 | 107. Bates D, Mächler M, Bolker B, Walker S. Fitting linear mixed-effects models using |
| 999 | lme4. arXiv:1406.5823. 2014 Jun 23 [cited 2023 Aug 23]. Available from: |
| 1000 | <https://arxiv.org/abs/1406.5823> |

1001

108.	Kuznetsova A, Brockhoff PB, Christensen RHB. lmerTest Package: Tests in Linear

| 1002 | Mixed Effects Models. J. Stat. Soft. [Internet]. 2017 Dec. 6 [cited 2023 Aug. 23];82(13):1- |
| --- | --- |
| 1003 | 26. Available from: <https://www.jstatsoft.org/index.php/jss/article/view/v082i13> doi: |
| 1004 | [10.18637/jss.v082.i13](http://doi.org/10.18637/jss.v082.i13) |
| 1005 | 109. Singmann H, Bolker B, Westfall J, Aust F, Ben-Shachar MS, Højsgaard S. afex: Analysis |
| 1006 | of Factorial EXperiments. Version 1.2-0 [software]. 2015 Oct 24 [cited 2023 Aug. 23]. |
| 1007 | Available from: <https://github.com/singmann/afex> |
| 1008 | 110. Eisenberg N, Strayer J, editors. Empathy and its development. Cambridge: Cambridge |
| 1009 | University Press; 1987. |
| 1010 | 111. Roberts E, Davies AD. Poor pitch singing: Response of monotone singers to a program of |
| 1011 | remedial training. Journal of Research in Music Education. 1975 Dec;23(4):227-39. doi: |
| 1012 | [10.2307/3344852](https://doi.org/10.2307/3344852) |
| 1013 | 112. Geringer JM. The relationship of pitch-matching and pitch-discrimination abilities of |
| 1014 | preschool and fourth-grade students. Journal of Research in Music Education. 1983 |
| 1015 | Jul;31(2):93-9. doi: [10.2307/3345213](https://doi.org/10.2307/3345213) |
| 1016 | 113. Yarbrough C, Green G, Benson W, Bowers J. Inaccurate singers: An exploratory study of |
| 1017 | variables affecting pitch-matching. Bulletin of the Council for Research in Music Education. |
| 1018 | 1991 Jan 1;107:23-34. Available from: <http://www.jstor.org/stable/40318418> |
| 1019 | 114. Demorest SM, Pfordresher PQ. Singing accuracy development from K-adult: A |
| 1020 | comparative study. Music Perception: An Interdisciplinary Journal. 2015 Feb 1;32(3):293- |
| 1021 | 302. doi: [10.1525/mp.2015.32.3.293](https://doi.org/10.1525/mp.2015.32.3.293) |

1022

115.	Welch GF, Sergeant DC, White PJ. Age, sex, and vocal task as factors in singing" in

| 1023 | tune" during the first years of schooling. Bulletin of the Council for Research in Music |
| --- | --- |
| 1024 | Education. 1997 Jul 1:153-60. |
| 1025 | 116. Bentley A. Measurement and development of musical abilities: Some research interests |
| 1026 | and findings. Journal of Research in Music Education. 1969 Apr;17(1):41-6. doi: |
| 1027 | [10.2307/3344182](https://doi.org/10.2307/3344182) |
| 1028 | 117. Brochard R, Dufour A, Després O. Effect of musical expertise on visuospatial abilities: |
| 1029 | evidence from reaction times and mental imagery. Brain Cogn. 2004 Mar;54(2):103-9. doi: |
| 1030 | [10.1016/s0278-2626(03)00264-1](https://doi.org/10.1016/s0278-2626(03)00264-1) |
| 1031 | 118. Jakobson LS, Lewycky ST, Kilgour AR, Stoesz BM. Memory for verbal and visual |
| 1032 | material in highly trained musicians. Music Perception. 2008 Sep 1;26(1):41-55. doi: |
| 1033 | [10.1525/mp.2008.26.1.41](https://doi.org/10.1525/mp.2008.26.1.41) |
| 1034 | 119. Patston LL, Tippett LJ. The effect of background music on cognitive performance in |
| 1035 | musicians and nonmusicians. Music Perception. 2011 Dec 1;29(2):173-83. doi: |
| 1036 | [10.1525/mp.2011.29.2.173](https://doi.org/10.1525/mp.2011.29.2.173) |
| 1037 | 120. Berkowska M, Dalla Bella S. Reducing linguistic information enhances singing |
| 1038 | proficiency in occasional singers. Ann N Y Acad Sci. 2009 Jul;1169:108-11. doi: |
| 1039 | [10.1111/j.1749-6632.2009.04774.x](https://doi.org/10.1111/j.1749-6632.2009.04774.x) |
| 1040 | 121. Hansen M, Wallentin M, Vuust P. Working memory and musical competence of |
| 1041 | musicians and non-musicians. Psychology of Music. 2013 Nov;41(6):779-93. doi: |
| 1042 | [10.1177/0305735612452186](https://doi.org/10.1177/0305735612452186) |

1043

122.	Jaschke AC, Honing H, Scherder EJA. Longitudinal Analysis of Music Education on

| 1044 | Executive Functions in Primary School Children. Front Neurosci. 2018 Feb 28;12:103. doi: |
| --- | --- |
| 1045 | [10.3389/fnins.2018.00103](https://doi.org/10.3389/fnins.2018.00103) |
| 1046 | 123. Lee YS, Lu MJ, Ko HP. Effects of skill training on working memory capacity. Learning |
| 1047 | and Instruction. 2007 Jun 1;17(3):336-44. doi: [10.1016/j.learninstruc.2007.02.010](https://doi.org/10.1016/j.learninstruc.2007.02.010) |
| 1048 | 124. Rickard NS, Vasquez JT, Murphy F, Gill A, Toukhsati SR. Benefits of a classroom based |
| 1049 | instrumental music program on verbal memory of primary school children: a longitudinal |
| 1050 | study. Australian Journal of Music Education. 2010;1:36-47. |
| 1051 | 125. Talamini F, Carretti B, Grassi M. The working memory of musicians and nonmusicians. |
| 1052 | Music Perception: An Interdisciplinary Journal. 2016 Dec 1;34(2):183-91. doi: |
| 1053 | [10.1525/mp.2016.34.2.183](https://doi.org/10.1525/mp.2016.34.2.183) |
| 1054 | 126. Cicchini GM, Arrighi R, Cecchetti L, Giusti M, Burr DC. Optimal encoding of interval |
| 1055 | timing in expert percussionists. J Neurosci. 2012 Jan 18;32(3):1056-60. |
| 1056 | [10.1523/jneurosci.3411-11.2012](https://doi.org/10.1523/jneurosci.3411-11.2012) |
| 1057 | 127. Matthews TE, Thibodeau JN, Gunther BP, Penhune VB. The Impact of Instrument- |
| 1058 | Specific Musical Training on Rhythm Perception and Production. Front Psychol. 2016 Feb |
| 1059 | 3;7:69. doi: [10.3389/fpsyg.2016.00069](https://doi.org/10.3389/fpsyg.2016.00069) |
| 1060 | 128. Wood A, Perez P, Ilari B, Habibi A. Longitudinal Evidence of Associations Between |
| 1061 | Music aptitude, Music Training and Cognitive Abilities in Childhood [Internet]. OSF; 2023. |
| 1062 | Available from: osf.io/wxp38 |
| 1063 | 129. Peplak J, Song JH, Colasante T, Malti T. "Only you can play with me!" Children's |
| 1064 | inclusive decision making, reasoning, and emotions based on peers' gender and behavior |
| 1065 | problems. J Exp Child Psychol. 2017 Oct;162:134-148. doi: [10.1016/j.jecp.2017.04.019](https://doi.org/10.1016/j.jecp.2017.04.019) |

1066

130.	Bamford JM, Davidson JW. Trait Empathy associated with Agreeableness and rhythmic

| 1067 | entrainment in a spontaneous movement to music task: Preliminary exploratory |
| --- | --- |
| 1068 | investigations. Musicae Scientiae. 2019 Mar;23(1):5-24. doi: [10.1177/1029864917701536](https://doi.org/10.1177/1029864917701536) |
| 1069 | 131. Cirelli LK, Einarson KM, Trainor LJ. Interpersonal synchrony increases prosocial |
| 1070 | behavior in infants. Dev Sci. 2014 Nov;17(6):1003-11. doi: [10.1111/desc.12193](https://doi.org/10.1111/desc.12193) |
| 1071 | 132. De Wied M, Maas C, Van Goozen S, Vermande M, Engels R, Meeus W, Matthys W, |
| 1072 | Goudena P. Bryant's empathy index. European Journal of Psychological Assessment. 2007 |
| 1073 | Jan;23(2):99-104. doi: [10.1027/1015-5759.23.2.99](https://doi.org/10.1027/1015-5759.23.2.99) |
| 1074 | 133. Oakley BFM, Brewer R, Bird G, Catmur C. Theory of mind is not theory of emotion: A |
| 1075 | cautionary note on the Reading the Mind in the Eyes Test. J Abnorm Psychol. 2016 |
| 1076 | Aug;125(6):818-823. doi: [10.1037/abn0000182](https://doi.org/10.1037/abn0000182) |
| 1077 | 134. Peterson E, Miller SF. The Eyes Test as a Measure of Individual Differences: How much |
| 1078 | of the Variance Reflects Verbal IQ? Front Psychol. 2012 Jul 5;3:220. doi: |
| 1079 | [10.3389/fpsyg.2012.00220](https://doi.org/10.3389/fpsyg.2012.00220) |
| 1080 | 135. Cho E. The relationship between small music ensemble experience and empathy skill: A |
| 1081 | survey study. Psychology of Music. 2021 May;49(3):600-14. doi: |
| 1082 | [10.1177/0305735619887226](https://doi.org/10.1177/0305735619887226) |
| 1083 | 136. Correia AI, Castro SL, MacGregor C, Müllensiefen D, Schellenberg EG, Lima CF. |
| 1084 | Enhanced recognition of vocal emotions in individuals with naturally good musical abilities. |
| 1085 | Emotion. 2022 Aug;22(5):894-906. doi: [10.1037/emo0000770](https://psycnet.apa.org/doi/10.1037/emo0000770) |
| 1086 | 137. Farmer E, Jicol C, Petrini K. Musicianship enhances perception but not feeling of |
| 1087 | emotion from others’ social interaction through speech prosody. Music Perception. 2020 Apr |
| 1088 | 11;37(4):323-38. doi: [10.1525/mp.2020.37.4.323](https://doi.org/10.1525/mp.2020.37.4.323) |

1089

138.	Weijkamp J, Sadakata M. Attention to affective audio-visual information: Comparison

| 1090 | between musicians and non-musicians. Psychology of Music. 2017 Mar;45(2):204-15. doi: |
| --- | --- |
| 1091 | [10.1177/0305735616654216](https://doi.org/10.1177/0305735616654216) |
| 1092 | 139. Swaminathan S, Schellenberg EG. Musical competence and phoneme perception in a |
| 1093 | foreign language. Psychon Bull Rev. 2017 Dec;24(6):1929-1934. doi: [10.3758/s13423-017-](https://doi.org/10.3758/s13423-017-1244-5) |
| 1094 | [1244-5](https://doi.org/10.3758/s13423-017-1244-5) |
| 1095 | 140. Swaminathan S, Schellenberg EG. Musical Competence is Predicted by Music Training, |
| 1096 | Cognitive Abilities, and Personality. Sci Rep. 2018 Jun 15;8(1):9223. doi: [10.1038/s41598-](https://doi.org/10.1038/s41598-018-27571-2) |
| 1097 | [018-27571-2](https://doi.org/10.1038/s41598-018-27571-2) |
| 1098 | 141. Schellenberg EG, Mankarious M. Music training and emotion comprehension in |
| 1099 | childhood. Emotion. 2012 Oct;12(5):887-91. doi: [10.1037/a0027971](https://doi.org/10.1037/a0027971) |
| 1100 | 142. Davidson JW. Bodily movement and facial actions in expressive musical performance by |
| 1101 | solo and duo instrumentalists: Two distinctive case studies. Psychology of Music. 2012 |
| 1102 | Sep;40(5):595-633. doi: [10.1177/0305735612449896](https://doi.org/10.1177/0305735612449896) |
| 1103 | 143. Cho E, Yeoun Han J. Small music ensemble and empathy: A replication study in a South |
| 1104 | Korean music student sample. Psychology of Music. 2022 Jul;50(4):1121-35. doi: |
| 1105 | [10.1177/03057356211031663](https://doi.org/10.1177/03057356211031663) |

<https://www.editorialmanager.com/pone/download.aspx?id=34042592&guid=3ed7a38f-5116-4f7d-88b0-1a0d86c38f11&scheme=1>

#### [Click here to access/download](https://www.editorialmanager.com/pone/download.aspx?id=34042586&guid=8bf568a9-581d-4634-9c0f-284fe50f1d8c&scheme=1)

[Supporting Information Supporting information.docx](https://www.editorialmanager.com/pone/download.aspx?id=34042586&guid=8bf568a9-581d-4634-9c0f-284fe50f1d8c&scheme=1)

[Click here to download Data Review URL http://osf.io/y6dfc/](http://osf.io/y6dfc/)
