# Supplementary material for: Long-term music instruction is partially associated with the development of socioemotional skills
Source: PLoS One. 2024 Jul 18;19(7):e0307373. doi: 10.1371/journal.pone.0307373 (PMC11257369; doi:10.1371/journal.pone.0307373)
Supplement: Attachment — Submitted filename: Response to Reviewers.docx [file pone.0307373.s006.docx]

**Response to Editor**

1. Please ensure that your manuscript meets PLOS ONE's style requirements, including those for file naming.

- We believe the manuscript meets the style requirements for PLOS ONE.

2. If you’ve not already done so, consider depositing your raw data in a repository to ensure your work is read, appreciated and cited by the largest possible audience.

- Our data has been uploaded to the Open Science Framework and is accessible at https://osf.io/y6dfc/.

3. Thank you for stating in your Funding Statement. Please also include the statement “There was no additional external funding received for this study.” in your updated Funding Statement.

- Our amended Funding Statement is as follows:

The Brain and Music Program (AH) at the Brain and Creativity Institute is supported by the GRoW at Annenberg Foundation (https://growannenberg.org/), the Los Angeles Philharmonic Association (https://www.laphil.com/), the Van Otterloo Family Foundation, and the National Endowment for the Arts (grant 1809970-38-18, https://www.arts.gov/). The funders had no role in study design, data collection and analysis, decision to publish, or preparation of the manuscript. There was no additional external funding received for this study.

4. Please include captions for your Supporting Information files at the end of your manuscript, and update any in-text citations to match accordingly.

- We have added captions for the supporting information located in the section titled “Supporting information” at the end of the manuscript.

5. Please review your reference list to ensure that it is complete and correct. If you have cited papers that have been retracted, please include the rationale for doing so in the manuscript text, or remove these references and replace them with relevant current references. Any changes to the reference list should be mentioned in the rebuttal letter that accompanies your revised manuscript. If you need to cite a retracted article, indicate the article’s retracted status in the References list and also include a citation and full reference for the retraction notice.

- We have updated the reference list with additional sources offered by the reviewers. Primarily, we have added references to explain concepts such as El Sistema and psychological constructs such as cognitive and affective empathy, theory of mind, and trait and state empathy. In addition, we have renumbered the in-text citations to match with the new list of references.

**Response to Reviewer 1**

Line 55: Consider defining what constitutes as “early childhood” for the less informed readers

- We’ve added UNESCO’s definition of early childhood (birth to eight years old).

Line 160: “In this paper, we refer to socioemotional skills as traits and capacities which contribute to an individual’s ability to maintain social relationships and respond to the emotions of others” - Include a citation given the general and/or current terminology misconceptions and variations. Suggestion: Kuther’s (2020) definition of socioemotional learning and development: “maturation of social and emotional functioning,” which would encompass your more specific definition. A more concise definition would be better, but I have seen this used before in music education literature. Kuther, T. L. (2020). Livespan development: Livesin context (2nd ed.). SAGE.

- Using the suggestion provided by the reviewer, we’ve included Kuther’s definition of socioemotional learning and development which we agree encompasses our conceptualization of socioemotional development.

Line 205: Please clarify “analyzed anonymously”

- We add that to analyze something anonymously means to access the data without any linking information to the participants’ identities.

Line 209: Change “is not relationship” to “was no relationship”

- Agreed. Text has been altered following the reviewer’s suggestion.

Line 222: cite “El Sistema” for readers who are interested in knowing more

- We’ve added the following additional resources on El Sistema:

Frega, A. L., & Limongi, J. R. (2019). Facts and counterfacts: A semantic and historical overview of El Sistema for the sake of clarification. *International Journal of Music Education*, *37*(4), 561-575.

Majno, M. (2012). From the model of El Sistema in Venezuela to current applications: learning and integration through collective music education. *Annals of the New York Academy of Sciences*, *1252*(1), 56-64.

Uy, M. S. (2012). Venezuela's national music education program El Sistema: Its interactions with society and its participants' engagement in praxis. *Music and Arts in Action*, *4*(1), 5-21.

Lines 237-243: Cite the method of providing stimulus back (Cohen or Rutkowski?)

- We clarify that stimuli were provided back not necessarily following either the Cohen or Rutkowski method. Instead, vocally trained experimenters sang the neutral syllable “la” to the participant, and asked the participant to respond using “la” in return.

Lines 237-243: Add a small description of the melodies (“5-9 note melodies that were major or minor”)

- Agreed. Melodies were clarified to have been 5-9 notes long in either major or minor modes.

Line 227: Omit “a” in “a soccer program” if it was also community-based

- Agreed. Text has been altered following the reviewer’s suggestion.

Line 268: revise to “”to which participants would then drum along.”

- Agreed. Text has been altered following the reviewer’s suggestion.

Line 295: revise to, “to which they must respond yes or no.”

- Agreed. Text has been altered following the reviewer’s suggestion.

Lines 389-400: Please include a sentence as to why d’s were not reported throughout.

- d’s were mistakenly unreported for group comparisons and have now been added throughout.

Line 440-441: “varied significantly” was confusing as a reader. Please rephrase language to align with other significant findings

- We rephrased “varied significantly” to “was significantly predicted by” to follow the reporting of other significant effects.

Lines 518-525: Consider making this its own paragraph

- Agreed, this section has been made its own paragraph.

Lines 522-523: This is the first mention that the children played viola and violin. Please include that detail in paragraph lines 220-225

- We’ve now included the requested information in the participants subsection.

Lines 524-525 implies that the children did not regularly sing in the El Sistema program. Consider mentioning around line 222 whether or not it’s known that students sing regularly, sometimes, or rarely in the program

- We clarified that students received about 2 hours of choir training each week during their first year.

Regarding lines 550-551: Studies have supported that shorter patterns are less challenging to reproduce for young singer

- The additional references offered by the reviewer have been incorporated into the paper.

Lines 602-607: Overreaching interpretation of the data. Consider omitting and allowing the reader to consider the variety of reasons why children in the sports program shared more than children in the music program (outside the scope of the current paper). INCREDIBLY interesting, however!

- Agreed. This section has been removed from the manuscript

**Response to Reviewer 2**

Too many keywords. Please consider writing specific keywords within the context.

- Agreed. We’ve changed the keywords to the following: El-sistema inspired program, middle childhood, longitudinal, pitch-matching, rhythmic entrainment, socioemotional development

“Rhythmic” entrainment in keywords

- Agreed. “Rhythmic” has been added for clarification.

Unclear whether or not systematic instruction in music theory, “technique”, etc. refers to instrumental playing or not

- We’ve added clarifying explaining systematic instruction refers to both instrumental and vocal training.

Line 44: The conceptual framework should have been written under specific subtitles that define related terms and concepts of the study

- We’ve restructured the paper to begin with a section explaining the conceptual framework of the paper. In addition, we’ve added additional subtitles for each primary construct of interest (e.g., Pitch-matching and rhythmic entrainment development, socioemotional development, etc.)

Lines 188-192: Besides the aim of your study, please consider writing your research questions explicitly in items. For instance, Research question …… etc.

- Agreed. We’ve explicitly included our research questions at the end of the introduction.

Line 193: Please explain and define the methodology clearly.

- We believe that the methodology is explained and defined clearly in the following subsections of the Methods section. We choose to include the phrase “Data and code for analysis are available at <https://osf.io/y6dfc/>” at the beginning of the Methods section following reporting conventions for psychology papers.

Line 221: “El Sistema” has to be described in concrete

- Agreed. Additional resources and information has been provided explaining the El Sistema approach.

Line 235: Please write this section in subtitles, e.g. Research instruments, Data collection procedure, Data analysis, etc.

- We have reworded our current subtitles “Measures” and “Procedure” to “Research instruments” and “Data collection procedure”, respectively.

Line 338: Data analysis should be described in the methodology section. In this section, consider writing the research outcomes of the analysis.

- We’ve restructured the paper so that the “Data analysis” section precedes “Results”.

Lines 705: Must be written in APA style, last version.

- We disagree and believe the citation conventions of PLOS ONE follow the Vancouver style.

**Response to Reviewer 3**

Line 23: What is the aim of this study ? What are the research questions? I would have appreciated a better understanding of the study's aims before learning about the population and the measurement tools used.

- Agreed. We include that our study aims to investigate the development of pitch-matching, rhythmic entrainment, and socioemotional development. We then continue explaining the study population and clarify the socioemotional skills of interest.

Line 111: I would have been interested to know the age of the participants in this study (as you presented the age of the children participating in the two studies cited above).

- We’ve added that Hove and Risen’s (2009) population included college-aged participants.

Line 118: In what context?

- We’ve added additional information explaining that Kirschner and Ilari’s (2014) spontaneous helping involved an experimenter accidentally dropping items and awaited a response from the participant. We’ve also included that the sharing task involved the experimenter implying that they’d like the participants to share their resources during a game.

Line 123: socioemotional skills such as…

- We’ve included “sharing and emotion-recognition” as socioemotional skills of interest potentially related to pitch-matching and rhythmic entrainment.

Line 153: Here, I wonder how old the population being studied in your study is (it was not clearly presented in the abstract... would it be a good idea to do so)?

- We’ve reworded the abstract to more explicitly state that the population was approximately 6.81 years old at baseline data collection.

Line 157: In this study, Brazilian children shared significantly fewer cookies with E2 than Brazilian children. E2 was from Germany. Does culture (or familiarity) have to be considered when studying prosocial behaviors? Would it be relevant to address this point when comparing those two studies?

- Although we agree that culture is important to consider when studying prosocial behaviors, we can’t be certain that the inconsistent findings between Study 1 and Study 2 are a result of culture and choose not to make that claim. The populations in the two studies are not directly comparable, as Study 2 is specifically noted to be highly diverse and not a single homogenous group. As such, the incongruent culture effect seen in Study 1 does not necessarily apply to Study 2 and would require additional research. However, we add that culture is an important contextual factor to consider.

Lines 159-161: I feel a bit lost ... I think that this section might have been presented earlier... Would it be relevant to present a conceptual framework in a section of the text? It would be necessary to refer to texts (articles, chapters) you have read to define this concept.

- Agreed. We’ve introduced a conceptual framework section at the beginning of the introduction to explain our constructs of interest more thoroughly.

Lines 163-165: Reference?

- We’ve added the following references for theory of mind, affective and cognitive empathy, and empathic responding, respectively:

Premack, D., & Woodruff, G. (1978). Does the chimpanzee have a theory of mind?. *Behavioral and Brain Sciences*, *1*(4), 515-526.

Chakrabarti, B., & Baron-Cohen, S. (2006). Empathizing: neurocognitive developmental mechanisms and individual differences. *Progress in Brain Research*, *156*, 403-417.

Stern, J. A., Botdorf, M., Cassidy, J., & Riggins, T. (2019). *Empathic responding and hippocampal volume in young children* (Vol. 55, No. 9, p. 1908). American Psychological Association.

Line 165: Add references

- We’ve added the following reference whose definition for socioemotional development we are following as a larger framework:

Kuther, T. L. (2022). *The Essentials of Lifespan Development: Lives in Context*. SAGE Publications.

Lines 168 and 174: This is not what you are measuring….

- Although we did not measure self-esteem in our study, we believe that it is an important construct to consider when relating socioemotional development and music training. We do, however, add and acknowledge that self-esteem is not measured in our study.

Lines 177-178: Would it be possible to be precise? Was this study about music education and prosociality - sharing or helping behaviors? This is what you are measuring…

- We add that Rickard, Bambrick, and Gill (2012) measured self-esteem, attitude towards school, social skills, depression, and more. Although not directly related to our study, we again, believe this history of investigating socioemotional skills and music education is important to consider.

Lines 188-190: This should be in the abstract.

- Agreed. This has been added to the abstract.

Line 196: How old were they at the moment of the data collection used in this study?

- We add clarifying language to state that participants were between 5.73 and 8.01 years old at baseline data collection.

Lines 198-200: Information presented in this sentence doesn't seem related. Could you present them independently?

- Agreed. The sentence has been separated and presented independently.

Line 219: During those years, did some of the children in the passive control group engage in sports programs? How did you know it?

- For all participants, we were aware of any additional extracurricular activities they engaged in through extensive parental interviews. As such, we know whether children in the passive control group engaged in sports or music programs, and if they did, discontinued participation in the study.

Line 232: N = ?

- N = 13 has been added

Line 233: N = ?

- N = 15 has been added

Line 255: Could you remind us what was the mean age of the participants at this time of the study?

- We believe that the mean age of participants was clearly stated in the methods section.

Line 257: Here again, I wonder what age are the children.

- We again believe that the participants’ ages were clearly stated in the methods section

Line 276: Why and how did you adapt the "dictator game"? How did you measure the validity of this tool following the adaptation?

- We added additional information explaining that this variation of the dictator game was previously used and validated by the following study:
- Flook L, Goldberg SB, Pinger L, Davidson RJ. Promoting prosocial behavior and self-regulatory skills in preschool children through a mindfulness-based Kindness Curriculum. Dev Psychol. 2015 Jan;51(1):44-51. doi: 10.1037/a0038256.

Line 289: This variable is not clearly defined previously.

- Agreed. We have now defined “empathic responding” in the introduction as the capacity to understand and appropriately respond to the emotional states of another person.

Line 289: This variable is not presented in the abstract and is not clearly defined previously.

- Agreed. We have now defined “theory of mind” in the introduction as the ability to recognize other individuals as having unique mental states.

Line 293: What is affective empathy? This has to be defined in the conceptual framework.

- Agreed. We have now defined “affective empathy” in the introduction as a form of empathy focused on the ability to recognize and resonate with the emotional state of another person.

Line 303: Please clearly define this concept previously. Empathy and sympathy need to be defined in the conceptual framework.

Line 305-306: I feel lost. Sympathy and empathy are two different concepts. Does this task measure sympathy, empathy, or both? Please clarify.

- Sympathy has been removed for clarity, the Index of Empathy is a measure for trait empathy.

Line 315: I feel a little bit lost. Were the following measures used to be sure that groups were equivalent? If yes, it would be important to remind the reader. If not, why did you use these measures?

- Measures for general cognitive ability were collected and used in analysis as covariates following the work done by Schellenberg, which found an association between cognitive ability and music ability. Clarification language has been added explaining this.

Lines 326-327: Idem

- I am unclear what this request means

Lines 333: Each year for seven years?

- Yes, data was collected each year for seven years.

Lines 343-344: I feel that this information should have been presented earlier.

- Agreed, this information has been reiterated in the introduction.

Line 347: Index of Empathy for Children and Adolescents

- I disagree with repeating the full name of the tool as the Index of Empathy was abbreviated previously

Table 1: rename variables to Sharing task, empathy, theory of mind, sympathy, cognitive affective empathy

- I disagree with renaming the measurements to the constructs they represent as listing the measurements themselves is more.

Lines 371-372: This might be presented in another paragraph.

- Agreed. This has been presented in its own paragraph.

Lines 378-380: Why?

- General cognitive ability has been found to predict music ability with work done by Schellenberg’s group, so we too include forms of general cognitive ability as covariats in our analysis.

Line 389: Full-Scale Intelligence Quotient

- I disagree with repeating the full name of the tool as this was abbreviated previously

Line 412: Did they score 0.62 points less or not? What do you mean by trend scoring 0.62 less?

Line 418: Idem

- I am unclear what this request means

Line 446: skills?

- Agreed.

Line 457: ?

- BIC stands for Bayesian Information Criterion which was abbreviated previously in the paper

Lines 566-567: And what could be the benefits of being in a more musical environment ...on parent-child relationships? ...on the child's musical development? ...on the child's non-musical development? Might you expand your idea a little bit more?

- Early exposure to music might jumpstart a child’s ability for basic fundamental musical skills. This has been expanded upon in this section.

Lines 603-604: Didn't you write before that children in the sport condition reveived ..

- I am unclear what this request means, nevertheless, this section has been removed with recommendations from another reviewer.

Line 700: Would you like to open up the discussion and briefly introduce the idea that participation in a non-formal music program might achieve different results?

- Agreed. Participation in a non-formal music program might offer different results as seen in work done by Rickard’s group. We add a brief sentence raising this issue and the need for additional research in this music education paradigm.
