# Supplementary material for: Long-term music instruction is partially associated with the development of socioemotional skills
Source: PLoS One. 2024 Jul 18;19(7):e0307373. doi: 10.1371/journal.pone.0307373 (PMC11257369; doi:10.1371/journal.pone.0307373)
Supplement: Attachment — Submitted filename: Response to Reviewers.docx [file pone.0307373.s007.docx]

**Response to Editor**

We have evaluated the works requested by reviewer 4 and have incorporated them into our manuscript.

**Response to Reviewer 4**

It is difficult to draw conclusions regarding music training effects from this study because children self-selected into training (i.e., predispositions are likely to have played a role), and the music training program was more intense (with more hours per week) than the control training.

- We agree that the interpretation for this study is difficult given selection bias and genetic predispositions. To remedy this, we re-emphasized the potential presence for these two in the discussion.

The title suggests a causal effect of music training on socioemotional skills that is not supported by the data. If I read the results correctly, for no measure the music group improved more over time than the controls groups. In fact, the opposite was found for sharing.

- We modified the title to clarify that the results of this study are not causal but represent associations. Nonetheless, the music group did improve in their emotion-matching ability relative to the sports group, so this point was made clearer in the results and discussion.

The Abstract needs a conclusion.

- The abstract was modified to include a conclusion and fit within the 300-word limit

There are several missing meta-analyses and reviews of the literature that are directly relevant for the framing and discussion of this study

- We’ve incorporated the recommended articles into the introduction and discussion, briefly covering music training’s association and lack thereof with transfer effects.
